# Stem cell–nanomedicine system as a theranostic bio-gadolinium agent for targeted neutron capture cancer therapy

**DOI:** 10.1038/s41467-023-35935-0

**Published:** 2023-01-18

**Authors:** Yen-Ho Lai, Chia-Yu Su, Hung-Wei Cheng, Chao-Yi Chu, Long-Bin Jeng, Chih-Sheng Chiang, Woei-Cherng Shyu, San-Yuan Chen

**Affiliations:** 1 Cell Therapy Center, China Medical University Hospital, Taichung, Taiwan; 2 College of Pharmacy, Taipei Medical University, Taipei, Taiwan; 3 Department of Materials Science and Engineering, National Yang Ming Chiao Tung University, Hsinchu, Taiwan; 4 Organ Transplantation Center, China Medical University Hospital, Taichung, Taiwan; 5 Graduate Institute of Biomedical Sciences, China Medical University, Taichung, Taiwan; 6 Neuroscience and Brain Disease Center, China Medical University, Taichung, Taiwan; 7 Translational Medicine Research Center, Drug development Center and Department of Neurology, China Medical University Hospital, Taichung, Taiwan; 8 Department of Occupational Therapy, Asia University, Taichung, Taiwan; 9 Frontier Research Centre on Fundamental and Applied Sciences of Matters, National Tsing Hua University, Hsinchu, Taiwan; 10 School of Dentistry, College of Dental Medicine, Kaohsiung Medical University, Kaohsiung, Taiwan

**Keywords:** Drug delivery, Nanoparticles, Radiotherapy, Targeted therapies

## Abstract

The potential clinical application of gadolinium-neutron capture therapy (Gd-NCT) for glioblastoma multiforme (GBM) treatment has been compromised by the fast clearance and nonspecific biodistribution of gadolinium-based agents. We have developed a stem cell–nanoparticle system (SNS) to actively target GBM for advanced Gd-NCT by magnetizing umbilical cord mesenchymal stem cells (UMSCs) using gadodiamide-concealed magnetic nanoparticles (Gd-FPFNP). Nanoformulated gadodiamide shielded by a dense surface composed of fucoidan and polyvinyl alcohol demonstrates enhanced cellular association and biocompatibility in UMSCs. The SNS preserves the ability of UMSCs to actively penetrate the blood brain barrier and home to GBM and, when magnetically navigates by an external magnetic field, an 8-fold increase in tumor-to-blood ratio is achieved compared with clinical data. In an orthotopic GBM-bearing rat model, using a single dose of irradiation and an ultra-low gadolinium dose (200 μg kg^−1^), SNS significantly attenuates GBM progression without inducing safety issues, prolonging median survival 2.5-fold compared to free gadodiamide. The SNS is a cell-based delivery system that integrates the strengths of cell therapy and nanotechnology, which provides an alternative strategy for the treatment of brain diseases.

## Abstract (web summary)

Gadolinium-neutron capture therapy (Gd-NCT) in glioblastoma shows promise but is limited by toxicity and short-half life in the brain. Here, the authors present a magnetised stem cell-nanoparticle system to facilitate brain penetrance of Gd-NCT and demonstrate its utility in an orthotopic rat glioblastoma model.

## Introduction

Glioblastoma multiforme (GBM), a grade IV glioma, is the most aggressive malignancy of the central nervous system because of inevitable recurrence after the current standard of care^1^. Recent studies have demonstrated that neutron capture therapy (NCT) can extend survival in GBM patients. For example, boron neutron capture therapy (B-NCT) using ^10^B-containing agents such as boronophenylalanine (BPA), sodium borocaptate (BSH), and polyhedral boranedianion GB-10 has been brought to clinical trials.

However, the efficacy of this approach has been compromised by the nonspecific biodistribution and rapid metabolism of the B-NCT agents^2,3^. The high-LET heavy particles produced by B-NCT have a traveling distance shorter than the diameter of a cell, thus requiring an homogeneously intratumoral distribution of boron agents to achieve therapeutic efficacy. However, tumor heterogeneity, the tumor microenvironment and solid stress inside the tumor would lead to non-uniform distribution of boron within tumor cells, reducing the potency of B-NCT^4–7^.

Gadolinium-neutron capture therapy (Gd-NCT) has been developed to address the weaknesses of B-NCT, including the short range and the lack of dose tracking^8–11^. Gadolinium (Gd)-agent provides the largest neutron capture cross-section, approximately 67-fold higher than that of ^10^B^7^. The gamma rays and internal convergent (IC) electrons emitted from Gd-NCT have stronger penetration ability than the alpha particles released in B-NCT, guaranteeing a more homogeneous energy deposition within the tumor^7,12^. Gd agents can also be used as T1-weighted magnetic resonance imaging (MRI) contrast agents to locate the tumor and track the dose distribution in real time. However, Gd agent presented a fast clearance (half-life approximately 2 h)^13,14^, necessitating a long infusion duration of up to several hours to achieve the required dose at the tumor^15–17^.

Nanotechnology has been implemented to improve the pharmacokinetic behavior of Gd agents and achieve an appropriate tumor-to-blood (T/B) ratio^10,18,19^. For example, Verry et al. reported that AGuIX, an ultra-small gadolinium-containing nanoparticle, showed strong tumor specificity to achieve radioenhancement in GBM treatment^20^. However, although the rapid metabolism guaranteed the safety of the nanoparticle, it would limit the application in Gd-NCT. Moreover, as the gamma radiation emitted during Gd-NCT has a longer radiation range, it is important to achieve deep tumor tissue penetration to reduce the possible adverse effects on adjacent cells. Therefore, an alternative strategy to simultaneously achieve penetration of the blood-brain barrier (BBB), GBM targeting, and tumor tissue penetration for enhanced Gd-NCT in GBM is urgently needed.

Mesenchymal stem cells derived from umbilical cord (UMSCs) can serve as a cellular vehicle to penetrate the BBB and achieve GBM-targeted delivery of Gd agents. For instance, Sonabend et al. used mesenchymal stem cells (MSCs) to deliver an oncolytic adenovirus to glioma and demonstrated a 46-fold increase in viral copies in tumor tissue^21^. However, direct internalization of Gd agent into UMSCs could lead to the dissociation of Gd^3+^ before the cells enter the target site^22,23^, causing toxicity to the UMSCs and impairing cellular function to reduce the efficiency of Gd delivery^24^.

**Fig. 1:**
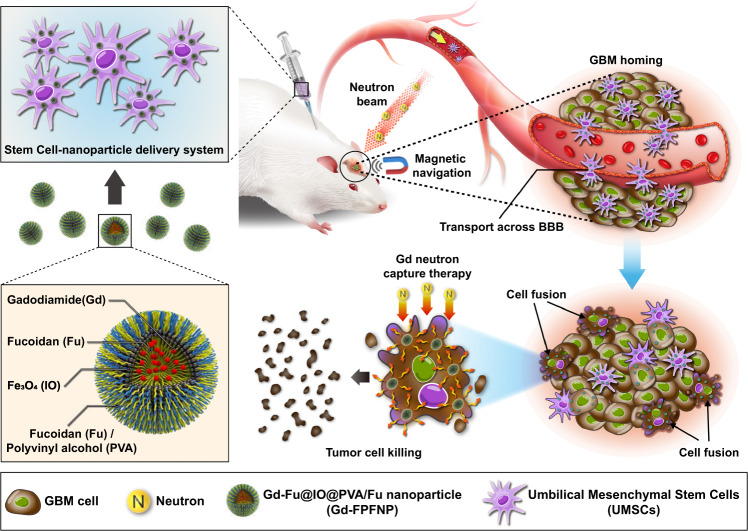
Mechanism of action for stem cell–nanoparticle system (SNS) in gadolinium-neutron capture therapy (Gd-NCT). Gd-Fu@IO@PVA/Fu nanoparticle (Gd-FPFNP) associated with umbilical cord mesenchymal stem cells (UMSCs) as a bio-NCT agent can cross the blood brain barrier (BBB) and fuse with tumor cells under magnetic navigation for enhanced neuron capture therapy.

In this work, in pursuit of an efficient and safe approach to Gd-NCT, we have integrated cell therapy and nanotechnology in a stem cell–nanoparticle system (SNS) to achieve a high T/B ratio. Figure 1 presents an illustration of the SNS and its mechanism of action to enhance Gd-NCT. Gd-Fu@IO@PVA/fucoidan nanoparticle (Gd-FPFNP) conceal the Gd agent and reduce cytotoxicity toward UMSCs during the journey toward homing and cancer cell fusion. Fucoidan (Fu) is a highly biocompatible material with anti-inflammatory activity^25^, which reduces oxidative stress via the Akt pathway and promotes nerve repair after radiotherapy^25,26^. The incorporation of the superparamagnetic iron oxide nanoparticles (IO) into Gd-FPFNP can magnetize the SNS and allow the real-time MR imaging along with magnetic guidance for enhanced locoregional accumulation at the tumor.

## Results

### Synthesis and characterization of Gd-FPFNP

**Fig. 2:**
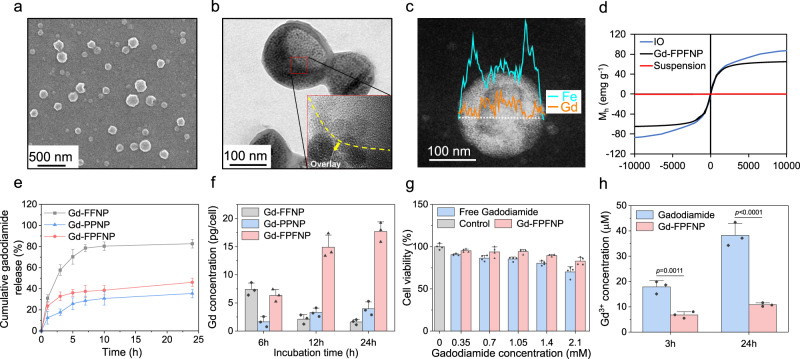
Characterization of gadodiamide (Gd)-loaded fucoidan-based nanoparticles. **a** SEM image of Gd-Fu@IO@PVA/Fu nanoparticle (Gd-FPFNP). **b** TEM image of Gd-FPFNP. Inset: high-resolution TEM image shows that superparamagnetic iron oxide nanoparticle (IO) was presented in the shell of Gd-FPFNP. **c** TEM line scan image and EDS analysis showed that Gd was encapsulated in the core of Gd-FPFNP. **d** The saturation of magnetization for IO and Gd-FPFNP was 84.7 and 73.5 emu g^-1^, respectively. **e** Cumulative gadodiamide release of Gd-Fu@IO@Fu nanoparticle (Gd-FFNP), Gd-PVA@IO@PVA nanoparticle (Gd-PPNP) and Gd-FPFNP in phosphate-buffered saline (PBS). The results were expressed as mean ± SD, *n* = 3 independent nanoparticles samples. **f** Gd concentration in umbilical cord mesenchymal stem cell (UMSC) after incubation with Gd-FFNP, Gd-PPNP and Gd-FPFNP for 6, 12, and 24 h (magnetic navigation was applied for 6 h). The results were expressed as mean ± SD, n = 4 independent UMSC samples. **g** Cell viability of UMSCs after the incubation with free gadodiamide and Gd-FPFNP for 24 h. The results were expressed as mean ± SD, *n* = 4 independent UMSC samples. The p values between free gadodiamide and Gd-FPFNP at the concentration of 0.35, 0.7, 1.05, 1.4 and 2.1 mM are 0.0053, not significant (ns, *p* > 0.05), 0.0025, 0.0007 and 0.0168, respectively. **h** Release of Gd^3+^ from free gadodiamide and Gd-FPFNP in culture medium. The results were expressed as mean ± SD, *n* = 3 independent nanoparticles samples. *P* values between free gadodiamide and Gd-FPFNP at 3 h and 24 h are 0.0011 and low than 0.0001, respectively. Statistical analysis in **g** and **h** was performed by Graph Pad Prism 9.0 Software using two-sided t-test compared with free gadodiamide.

The morphology of Gd-FPFNP was observed using scanning electron microscopy (SEM) as shown in Fig. 2a. The core-shell structure of Gd-FPFNP was visualized using transmission electron microscopy (TEM), in which the collapsed shell was overlaid and displayed a dark contrast as shown in the inset high-resolution image in Fig. 2b. Elemental mapping imaging with line scanning profile using energy-dispersive X-ray spectroscopy (EDS) demonstrated that the iron (Fe) signal peaked at the two ends whereas the Gd signals were centralized in the core (Fig. 2c). These results demonstrated that IOs were distributed in the shell of Gd-FPFNPs while gadodiamide was concealed in the core.

Fourier-transform infrared spectroscopy (FTIR) was used to confirm the existence of Fu in Gd-FPFNP. The characteristic peaks for Fu are S=O antisymmetric stretching vibration of the sulfate group and C–O–S bond where the sulfate ester is bound to the fucose, located at 1253 cm^−1^ and 842 cm^−1^, respectively (Fig. S1). These signals were also present in the Gd-FPFNP groups, indicating that Fu was on the surface of Gd-FPFNP. Saturated magnetization of IO and Gd-FPFNP was measured as 84.7 and 73.5 emu g^−1^, respectively, demonstrating that Gd-FPFNP retained magnetic properties after synthesis (Fig. 2d).

Gadodiamide-loaded nanoparticles synthesized using fucoidan (Gd-Fu@IO@Fu nanoparticle, Gd-FFNP) or PVA (Gd-PVA@IO@PVA nanoparticle, Gd-PPNP) alone were used as controls for comparison with FPFNPs (Fig. S2). The heterogeneous size distribution was also noted using dynamic light scattering (DLS), which showed a wide size band (Fig. S3a). The size and polydispersity index (PDI) of Gd-FPFNP, Gd-PPNP, and Gd-FFNP was 220 nm/0.146, 190 nm/0.131, and 385 nm/0.305, respectively. The smaller PDI of Gd-FPFNP compared to Gd-FFNP might be attributed to the formation of hydrogen bonds between PVA and Fu. Compared with the neutral zeta potential of Gd-PPNP, both Gd-FFNP and Gd-FPFNP exhibited a strongly negative-charged surface due to the presentation of sulfate groups in Fu (Fig. S3b). The stability of Gd-FPFNP, Gd-FFNP, and Gd-PPNP in PBS and PBS solution containing 10% serum albumin (simulating the stability of nanoparticles in blood) was evaluated by measuring the size change using DLS (Fig. S4)^27^. Only Gd-FPFNP maintained a monodispersed condition at day 28 in both solutions and provided steric hindrance to prevent protein opsonization and allow it to remain intact in physiological conditions^28^.

### Production and screening for the ideal SNS formulation

The Gd content in the various Gd-loaded nanoparticles was measured using inductively coupled plasma mass spectrometry (ICP-MS). As shown in Table S1, Gd-FPFNP loaded the highest amount of gadodiamide, showing an encapsulation efficiency (EE) of 59.64% and loading capacity (LC) of 28.57%. Gd-FFNP was structurally unstable (Figure S2) and showed 17.9% less loaded gadodiamide compared with Gd-FPFNP (13.7 mg vs. 16.69 mg, Table S1). The 24-h release profiles of Gd-FPFNPs and Gd-PPNP shown in Fig. 2e reveal a slow-release pattern, with cumulative release of gadodiamide stabilizing at 46% and 35%, respectively. The slow-release behavior was due to the distribution of the dense PVA chains within the shell, which created a diffusion barrier to conceal Gd in the core (Fig. 2c). In contrast, Gd-FFNP displayed a fast gadodiamide release rate resulting from its unstable structure, with more than 70% of the gadodiamide released within 5 h.

The cell morphology and biological properties of UMSCs obtained from umbilical cord Wharton’s jelly presented the same surface markers as bone marrow MSCs (Fig. S5)^29^. As the therapeutic efficacy of Gd-NCT relies on the amount of Gd agent accumulated at GBM, the internalization efficiency of Gd-FPFNP, Gd-FFNP, and Gd-PPNP into USMCs was investigated from 6 to 24 h. The Gd-FFNP-treated UMSC group displayed the highest Gd content at 6 h incubation compared with the other groups (Fig. 2f). However, the Gd content significantly decreased at 12 and 24 h incubation, representing low retention ability of gadodiamide in this formulation. The result is consistent with the fast gadodiamide release behavior shown in Fig. 2e. Encouragingly, the Gd content in the UMSCs treated with Gd-FPFNP steadily increased from 6 to 24 h, displaying the highest concentration compared with the other groups at 24 h incubation. The substantially enhanced gadodiamide accumulation was attributed to both the slow gadodiamide release behavior and the enhanced cell uptake promoted by the Fu-coated surface of Gd-FPFNP^30,31^. Thus, Gd-FPFNP-treated UMSCs with the highest Gd content and stability were selected as the stem cell–nanoparticle system (SNS) for further studies.

### Characterization of SNS

The internalization of Gd-FPFNP into UMSCs was observed using TEM. Figure S6 shows that the internalized Gd-FPFNP in USMC accumulated in the cytoplasm at 24 h of incubation. The interaction between Gd-FPFNP and UMSCs over time was investigated using confocal laser-scanning microscopy (CLSM) and flow cytometry (Fig. S7a, b) from 6 h to 5 days. Quantum dots (QD) were used to label Gd-FPFNP to facilitate the observation. It was noted that the fluorescence intensity reached a plateau between 12 and 24 h and decreased after 48 h, most likely due to cell division and partial exocytosis. At 24 h, UMSCs demonstrated a 91% stronger fluorescence intensity compared with the control group (Fig. S7b). Thus, the optimal incubation time for UMSCs was between 12 h and 24 h.

The additional application of an external magnetic field significantly increased cellular uptake of Gd-FPFNP from 6 to 24 h incubation (Fig. S8a). When treated with an equivalent Gd concentration, free gadodiamide had the lowest intracellular Gd content due to inefficient cell internalization (Fig. S8b). In contrast, the Gd-FPFNP group showed a higher intracellular Gd concentration, demonstrating the ability to deliver and retain gadodiamide in UMSCs. The application of the magnetic field significantly enhanced gadodiamide accumulation in UMSCs, showing a 2.3-fold increase compared to the group without MN.

As Gd^3+^ ions released from gadodiamide might affect the UMSCs during the transport of Gd-FPFNP to GBM, it was necessary to assess the cytotoxicity and perform functional assays after Gd-FPFNP internalization. Gd-FPFNP-internalized UMSCs showed a higher cell viability at 12 h incubation compared with those exposed to free gadodiamide in the equivalent Gd range from 0.35 to 2.1 mM (Fig. 2g). To assess the release of Gd^3+^ ions from Gd and Gd-FPFNP, we measured the amount of Gd^3+^ ions in PBS using xylenol orange agent^32^. The concentration of Gd^3+^ ions released from free gadodiamide was 38.6 μM at 12 h incubation, which is similar to the published literature (Fig. 2h)^33^. In contrast, the concentration for Gd-FPFNP was much lower, 10.8 μM at 12 h, indicating that Gd-FPFNP effectively concealed gadodiamide in the core to prevent its dissociation into Gd^3+^ and thus enhanced the biocompatibility of Gd-FPFNP toward UMSCs. In this assessment, the SNS formulation containing Gd-FPFNP loaded with 1.05 mM gadodiamide showed favorable biocompatibility and was selected for further studies.

The proliferation and migration of UMSCs after co-culture with Gd-FPFNP for 12 h were assessed using BrdU assay and a transwell migration assay, respectively. The results revealed that application of an external magnetic field and internalization of Gd-FPFNP did not affect the proliferation and migration of SNS compared to UMSCs alone (Fig. S9a, b). Furthermore, the SNS retained multipotent differentiation potential as characterized using adipogenic, chondrogenic, vascular tube, and osteogenic differentiation formation assays (Fig. S9c). SNS can also differentiate into neuroglial cells upon appropriate treatment, presenting an extended neurite-like morphology arranging into a network and positive expression of MAP-2, Tuj-1, and GFAP by immunofluorescent staining (Fig. S9d). We also examined the expression of major multi-potency markers on SNS using flow cytometry and demonstrated that SNS maintained surface expression of HLA-DR (<2%), CD11b (<2%), CD19 (<2%), CD34 (<2%), CD45 (<2%), CD73 (>95%), CD90 (>95%), and CD105 (>95%) at levels similar to those of control UMSCs (Fig. S10). Our results thus demonstrated that the SNS sustained UMSC properties to facilitate Gd agent delivery to GBM.

### SNS is a potential MRI contrast agent for achieving real-time tracking

As gadodiamide and IO are both MRI contrast agents, SNS can be tracked in vivo with a dual-imaging strategy. MR relaxation rates (r_1_) and (r_2_) were measured by a relaxometer (0.5 Tesla, 20 MHz, 37 °C). The r_1_ values of gadodiamide and Gd-FPFNP were 4.0 and 17.9 mM^−1^s^−1^, respectively (Fig. S11a). The *r*_1_ value significantly increased with Gd-FPFNP compared to free gadodiamide. The *r*_2_ value of FPFNP (166.0 mM^−1^s^−1^) was slightly lower than that of Gd-FPFNP (202.9 mM^−1^s^−1^), implying that the interference effect of gadodiamide on the *r*_2_ value is minor (Fig. S11b). The high r_1_ value and lower r_2_/r_1_ ratio in the Gd-FPFNP suggest that Gd-FPFNP could be a qualified contrast agent^34^. T1- and T2-weighted images (T1WI and T2WI) were used to evaluate the signals in SNS and UMSCs treated with free gadodiamide (U-Gd) in vitro. SNS showed significantly stronger intensity on both T1WI and T2WI, demonstrating that the association with Gd-FPFNP was much more effective than free gadodiamide alone (Fig. S12).

### Dose and administration route of SNS

To optimize the SNS treatment, the dose and the route of administration were examined in a luciferase-expressing F98 (F98-Luc) orthotopic rat model using MRI. Among the three cell doses delivered via intracarotid injection, administration of 2 × 10^6^ cells demonstrated the clearest contrast of GBM margin compared with the pre-contrast MRI image (Fig. S13), indicating adequate accumulation of Gd in the tumor. In addition, we compared the delivery efficiency of Gd-FPFNP and SNS administrated via different routes (intracarotid injection or intravenous injection) with or without magnetic navigation (MN) using MRI. To facilitate MN, a magnet (5 mm in diameter, 0.5 mm in height, 0.5T) was placed on the external side of the skull for 24 h.

A well-marginated and homogenous mass with strong hyperintense signals located over the striatal area of the tumor was observed for SNS delivered by intracarotid injection (lane 2, Fig. S14). Delivery of SNS by intravenous injection resulted in a less hyperintense signal with an irregular margin in the GBM region compared to intracarotid injection (lane 3, Fig. S14). The reduced accumulation in the intravenously injected group might be attributed to the first-pass effect of lung entrapment upon administration. Although Gd-FPFNP was not expected to cross the BBB, we observed an enhanced contrast signal at the GBM area under MN (lane 1, Fig. S14). These results could be attributed to partial destruction of the BBB by GBM^35,36^. However, these signals were randomly distributed within the brain, indicating a lack of specificity to the tumor. Therefore, intracarotid administration of SNS, which resulted in specific and increased accumulation at the tumor, was adopted in this study.

### GBM homing features of SNS

UMSCs have been reported to possess tumor-homing activity^37–39^. The CXCR4 and CCR2 expressed on UMSCs can interact with overexpressed SDF-1α and MCP-1 in GBM^40–42^, thus providing tumor-homing tropism^38,43,44^. Moreover, after activation by SDF-1α, VLA-4 on the surface of UMSCs can interact with VCAM-1 adhesion molecules and β1 integrin on endothelial cells of the BBB and aid penetration of the barrier by rolling and migration to achieve intracranial Gd agent delivery^45–47^.

We examined the SDF-1α expression level in GBM-bearing and healthy rats (Fig. S15a) and found that SDF-1α mRNA levels in tumor-infiltrated brains were significantly higher than in normal brains (Fig. S15b). The abundance of SDF-1α in the microenvironment can promote penetration of the BBB by SNS. We further confirmed that SNS was able to cross the BBB and localize in the brains of F98-bearing rats (Fig. S16a). In contrast, SNS did not accumulate in the brains of healthy mice, even under application of MN. Moreover, as a control group, Gd-FPFNP-treated 4T1 cells (4T1-Gd-FPFNP) that do not respond to SDF-1α did not accumulate at brain tumors under IVIS. ICP-MS was then used to measure the Gd concentration in GBM and normal brain tissues for evaluating the homing ability of SNS. As GBM is with highly infiltrative nature, it diffuses quickly and causes swelling in the inoculated hemisphere of brain. Therefore, we separated the brain into right and left contralateral hemisphere, where the right hemisphere inoculated with F98 tumor cells was identified as the tumor-infiltrated hemisphere; in contrast, the left brain without tumor invasion was defined as the normal tissue. As shown in Fig. S16b, we did not observe Gd accumulation in the sham group. In contrast, SNS groups presented a higher Gd concentration in both sides of the brain when compared with 4T1-Gd-FPFNP. Of note, there was no statistical difference between the left (i.e., normal tissue) and right brain (i.e., tumor) of the 4T1-Gd-FPFNP group. Encouragingly, SNS and SNS plus MN group demonstrated a significantly higher Gd level in right brain (i.e., tumor site), indicating that SNS possessed the affinity toward GBM and showed the homing capability to increase Gd content in tumor (Fig. S16b).

### SNS targets and fuses with GBM cells

**Fig. 3:**
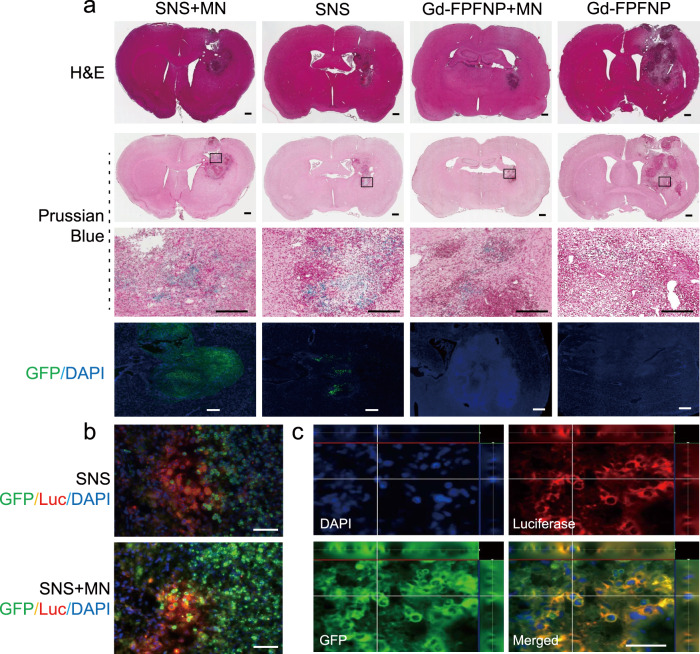
Distribution of stem cell–nanoparticle system (SNS) and Gd-Fu@IO@PVA/Fu nanoparticle (Gd-FPFNP) in the tumor tissues of F98 tumor-bearing rats. **a** H&E staining (lane 1) and Prussian blue staining (lane 2) of the brains at 24 h post treatment with SNS plus magnetic navigation (MN), SNS, Gd-FPFNP plus MN, and Gd-FPFNP. Scale bar = 500 μm. GBM area framed in the in lane 2 was magnified in the high-power field (lane 3, scale bar = 500 μm). Lane 4 was the fluorescent staining of same region (GFP represented SNS, DAPI represented nuclei, scale bar = 50 μm). The study was performed with *n* = 3 rats. The images in lanes 1–4 are representative of at least 3 other images. GFP green fluorescent protein. **b** Immunofluorescent images of tumor tissues from luciferase-expressing F98 (F98-Luc)-tumor-bearing rats. The rats were treated with SNS plus MN or SNS alone. UMSCs were modified with GFP. Nuclei were stained using DAPI. Luc luciferase. Scale bar = 50 μm. The study was performed with *n* = 3 rats and the images are representative of 4 images. **c** Colocalization of GFP^+^ UMSCs (green) with F98-Luc cells (red) over the striatal area at 24 h post injection of SNS with MN in the F98-Luc GBM rat model. Scale bar = 50 μm. The study was performed with *n* = 3 rats and the images are representative of 4 images.

The trafficking of SNS and Gd-FPFNP after BBB penetration was monitored using multiple assays. In histological H&E staining, tumor regions with clear necrosis margins were observed in each treatment group (Fig. 3a, first lane). The IO in Gd-FPFNP and SNS can be stained using Prussian blue. The Prussian blue signals in tumors were stronger in the SNS groups than with Gd-FPFNP (lane 2 and 3 in Fig. 3a and Table S2), indicating that SNS was more effective in delivering Gd agents to GBM. This result was consistent with the MRI images in Fig. S15.

To facilitate observation at the cellular level, SNS was modified with green fluorescent protein (GFP) for immunohistochemical staining. SNS with GFP signal was observed in the tumor region, especially for SNS plus MN group. In contrast, Gd-FPFNP groups free of UMSCs did not present the signal (lane 4, Fig. 3a). Moreover, the GFP signal from the SNS overlaid with the luciferase (Luc) signal from GBM (F98-Luc), indicating that SNS was able to fuse with GBM cells within 24 h post intracarotid administration (Fig. 3b). Results from confocal microscopic imaging demonstrated that GFP^+^ fluorescent cells colocalized with l F98-Luc tumor cells over the striatal area (Fig. 3c). The percentage of colocalization was quantified at 38.4 ± 1.7% using Image J (scored as the ratio of double-positive cells over total cell number under 10 high-power fields). These findings demonstrated SNS can migrate to the microenvironment of GBM and fuse with the tumor cells after BBB penetration, precisely delivering Gd agents to the site of interest.

### Biodistribution and T/B ratio of SNS

**Fig. 4:**
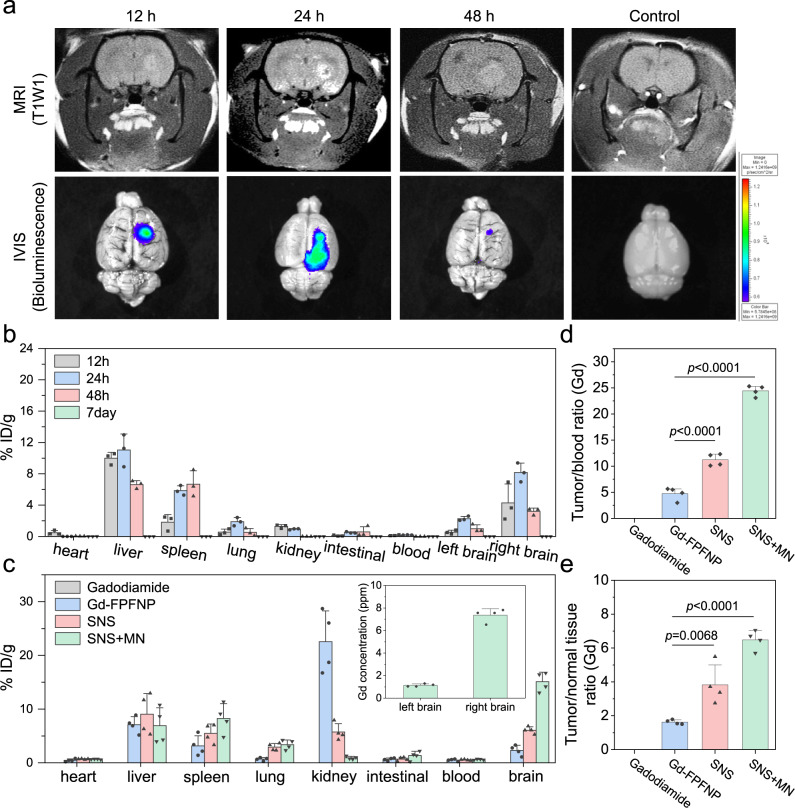
Tumor accumulation and biodistribution of stem cell–nanoparticle system (SNS) and Gd-Fu@IO@PVA/Fu nanoparticle (Gd-FPFNP). **a** Magnetic resonance imaging (MRI) and in vivo imaging system (IVIS) images of the F98 glioblastoma multiforme (GBM) at different times. The study was performed with *n* = 6 rats. The images for MRI and IVIS are representative of 6 images. **b** Gd concentration in various organs at post 12 h, 24 h, 48 h, and 7d intracarotid injection of SNS. The Gd content was measured suing inductively coupled plasma mass spectrometry (ICP-MS). The results were expressed as mean ± SD, *n* = 3 rats. % ID/g: percentage of injected dose per gram of tissue. **c** Gd concentration in various organs at 24 h post treatment of gadodiamide, Gd-FPFNP, SNS, and SNS plus magnetic navigation (MN). The concentrations for gadodiamide group were all below the detection limit. The results were expressed as mean ± SD, *n* = 4 rats. **d** Tumor-to-blood (T/B) ratio and **e** Tumor-to-normal tissue (T/N) ratio of gadodiamide, Gd-FPFNP, SNS, and SNS plus MN groups at 24 h post treatment. The results were expressed as mean ± SD, *n* = 4 rats. Statistical analysis in **d** and **e** were performed by Graph Pad Prism 9.0 Software: one-way ANOVA with Tukey’s multiple comparisons test. For **d**, the p values of Gd-FPFNP to SNS and SNS plus MN are both are <0.0001. For **e**, the *p* values of Gd-FPFNP to SNS and SNS plus MN are 0.0068 and <0.0001, respectively.

The accumulation of SNS in GBM was analyzed using MRI and the in vivo imaging system (IVIS) at 12 h, 24 h, and 48 h post intracarotid injection. The tumor signal of Gd agent in T1WI-MRI peaked at 24 h post injection (first lane in Fig. 4a). Similarly, SNS with luciferase expression can be monitored using IVIS, which exhibited a significantly stronger signal in the tumor at 24 h post injection (second lane in Fig. 4a and Fig. S17).

Accurate quantification of Gd content in vital organs was further performed using ICP-MS at 12 h, 24 h, 48 h, and 7d post administration. Consistent with the MRI and IVIS results, intratumoral Gd content of the right brain (where GBM cells were inoculated) reached a plateau at 24 h post administration of SNS, indicating the ideal timing to perform NCT (Fig. 4b). Moreover, the Gd content in the tumor-infiltrated right brains was significantly higher than in the normal left brain, indicating that SNS exhibited a GBM homing effect. Other than the brain, Gd was shown to be mainly distributed in the liver and kidney, but this was cleared within 7 days, leaving less than 10 ppb of Gd detected in all organs.

The biodistribution of gadodiamide alone, Gd-FPFNP, SNS, and SNS at 24 h post intracarotid injection was also studied using ICP-MS (Fig. 4c). Gd content was not detected in most organs for the rats treated with free gadodiamide, reflecting the fast clearance of gadodiamide in vivo. In contrast, Gd could be detected in most organs for both Gd-FPFNP- and SNS-treated groups, indicating increased stability and retention time of gadodiamide in such formulations. Significantly reduced Gd content in kidney and enhanced Gd content in brain were identified in the SNS group when compared to the Gd-FPFNP group. The results address the advantages of using SNS to overcome the limitations of BBB penetration and GBM localization that have hampered previous drug delivery systems. Moreover, combining SNS with MN can achieve significantly higher accumulation of Gd in GBM (15.4 %ID/g, or 4.5 ppm) compared to SNS without MN (6.07 %ID/g, or 2.01 ppm).

The tumor-blood (T/B) ratio of SNS was 11.05, which was 2.32-fold higher than that of Gd-FPFNP (Fig. 4d). These results were consistent with the semiquantitative data shown in Fig. 3 and Table S2, in which Gd-FPFNP was randomly distributed in the brain but not directly localized in the tumor. Moreover, SNS plus MN showed a T/B ratio of 24.4, which was 2.22-fold and 5.19-fold higher than that of SNS and Gd-FPFNP, respectively. The high T/B ratio of SNS plus MN demonstrates a significant improvement compared to prior studies, which reported a T/B ratio of approximately 2.5^10^.

In addition to T/B ratio, the tumor-to-normal-tissue (T/N) ratio, which can be used to evaluate the potential risks to the adjacent tissues, was assessed. The tumor and healthy tissues in the brain (Fig. S15, red box) were carefully separated for measurement of Gd content to calculate the T/N ratio. The T/N ratio of SNS and SNS with MN was 3.83 and 6.46 respectively, which was 2.1 and 3.5-fold higher than that of the Gd-FPFNP group (Fig. 4e). These results indicated that SNS showed preferential accumulation at the tumor, and that MN could further effectively promote the tumor localization effect.

### SNS enhanced the therapeutic effect of Gd-NCT and prolonged survival time

Prior to performing the in vivo animal experiment, the therapeutic effect of the SNS-derived NCT on GBM8401 cells was assessed in vitro. The SNS was co-cultured with GBM8401 cells for 24 h under MN before NCT, and the cell viability was be measured at 24 h post neutron irradiation. At 24-h co-culture, SNS was able to fuse with GBM8401, demonstrating the colocalization of two nuclei in one cell (Fig. S18). As shown in Fig. S19, after NCT, SNS demonstrated a superior tumoricidal effect on GBM8401 compared with free Gd, which was barely internalized into tumor cells^48^.

**Fig. 5:**
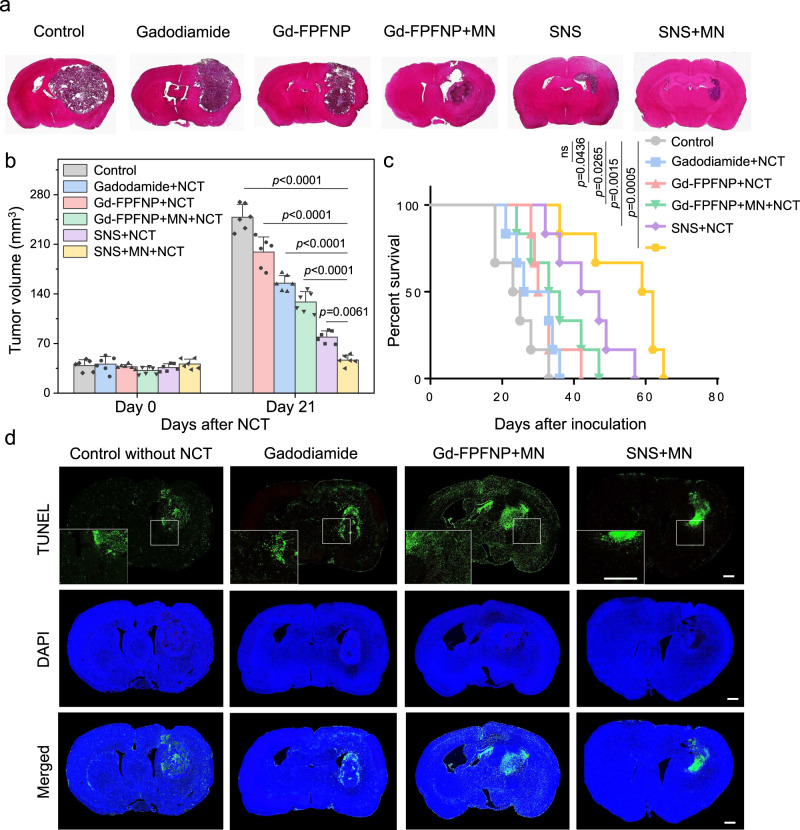
Therapeutic efficacy and safety assessment of GBM-bearing rats after various gadolinium-neutron capture therapy (Gd-NCT). **a** H&E staining of the brains for the GBM-bearing rats at 21 days post Gd-NCT in different groups. The study was performed with *n* = 6 rats. The images of H&E stain are representative of 6 images. **b** Tumor volumes for different groups before and after neutron capture therapy. The results were expressed as mean ± SD, *n* = 6 rats. Statistical analysis was performed by Graph Pad Prism 9.0 Software; one-way ANOVA with Tukey’s multiple comparisons test. The *p* values of SNS plus MN and NCT to control, Gadodiamide plus NCT, Gd-FPFNP plus NCT, Gd-FPFNP plus MN and NCT, SNS plus NCT are <0.0001, <0.0001, <0.0001, <0.0001, and 0.0061, respectively. **c** Survival study for GBM-bearing rats (*n* = 6) treated with saline, gadodiamide plus NCT, Gd-Fu@IO@PVA/Fu nanoparticle (Gd-FPFNP) plus NCT, Gd-FPFNP plus magnetic navigation (MN) and NCT, stem cell–nanoparticle system (SNS) plus NCT, and SNS plus MN and NCT. A Kaplan–Meier survival analysis was performed and reported as median survival times with 95% confidence interval. The statistical difference between the six conditions was determined by log-rank analysis. The *p* values of control group to Gadodiamide plus NCT, Gd-FPFNP plus NCT, Gd-FPFNP plus MN and NCT, SNS plus NCT, and SNS plus MN and NCT are not significant (ns, p  >  0.05), 0.0436, 0.0265, 0.0015, and 0.0005, respectively. **d** TdT-mediated dUTP Nick-End Labeling (TUNEL) staining of brain tissue post treatments. Cell nuclei (blue), TUNEL-positive cells (green). Scale bar = 500 µm. The study was performed with *n* = 6 rats. The TUNEL images are representative of 6 images.

The in vivo therapeutic study was performed using a F98-Luc orthotopic GBM rat model. In the untreated control group, the tumor had infiltrated through the whole right brain and invaded across the midline at 21 days after tumor inoculation (Fig. 5a), demonstrating the aggressive nature of GBM. Following the treatment schedule shown in Fig. S20a, treatment with UMSCs alone (without Gd agent) or UMSCs plus NCT was not able to attenuate the tumor progression compared with the control group (Fig. S20b, c), indicating that without the presence of Gd agents, neutron irradiation or UMSCs cannot offer therapeutic benefits.

Treatment with free gadodiamide with NCT slightly inhibited the tumor progression (Fig. 5a, b), but failed to significantly extend the median survival. Both formulated gadodiamide in Gd-FPFNP and SNS demonstrated a much stronger antitumor effect under NCT, significantly inhibiting tumor invasion and progression at 21 days post tumor inoculation (Fig. 5b). The antitumor activity of SNS outperformed that of Gd-FPFNP in terms of local control (Fig. 5a), tumor volume suppression (Fig. 5b), and median survival (Fig. 5c). The additional application of MN further promoted the therapeutic efficacy of Gd-FPFNP and SNS. With only one treatment, SNS plus MN achieved superior local tumor control (Fig. 5a), significantly extending the median survival to 59 days compared with 23, 30, 33, and 42 days for control, Gd-FPFNP, Gd-FPFNP plus MN, and SNS, respectively (Fig. 5c).

To understand the affected brain region and the safety profile after NCT, we performed a whole-brain TUNEL assay to evaluate the outcome of the various treatments. The groups treated with free gadodiamide showed a negligible difference in apoptosis compared to the control group. Gd-FPFNP plus MN induced stronger apoptosis around the tumor, showing a superior tumoricidal effect compared with free gadodiamide. However, both formulations induced a large area of apoptosis, indicating nonspecific distribution within the brains resulting from the lack of targeting ability (Fig. 5d). In contrast, the group treated with SNS plus MN showed localized cell death within the tumor after NCT, leaving the adjacent healthy brain tissues unharmed. The body weight of the rats was also monitored throughout the entire experiments. As shown in Fig. S21, the rapid tumor progression in control group has significantly affected the rats and caused a substantial decrease in body weight. In contrast, the body weight for SNS and SNS plus MN groups was steady, indicating that the tumors were effectively suppressed by the treatments while no obvious systemic toxicity was induced after the irradiation. The results demonstrated that SNS targeted and fused with GBM, restricting the deposition of gamma rays emitted from Gd-NCT to within the tumor and augmenting the therapeutic index of the treatments^12,49^.

The exposure time used in the study was determined based on the principle of clinical radiotherapy – to achieve an adequate/maximum dose at tumor without irreversibly damaging the organ-at-risk (OAR). The internal dosimetry was designed dependent on several factors including the localized Gd agents at the target tissues, the dose limitation of the OAR, and the T/B ratio. All the factors were collected and analyzed using Monte Carlo N-particle transport code to generate the internal dosimetry and treatment plan, which could be further translated into the NCT exposure intensity and time. The final internal dose for the SNS with MN group was calculated to be 5.846 Gray (Gy) per fraction NCT^50^, which is higher than one regular fraction dose in clinical use (1.5–2 Gy). This dose can cause DNA damage in the tumor cell and provided adequate deposition of radiation within the tumor^51^.

### Safety assessment of SNS

**Fig. 6:**
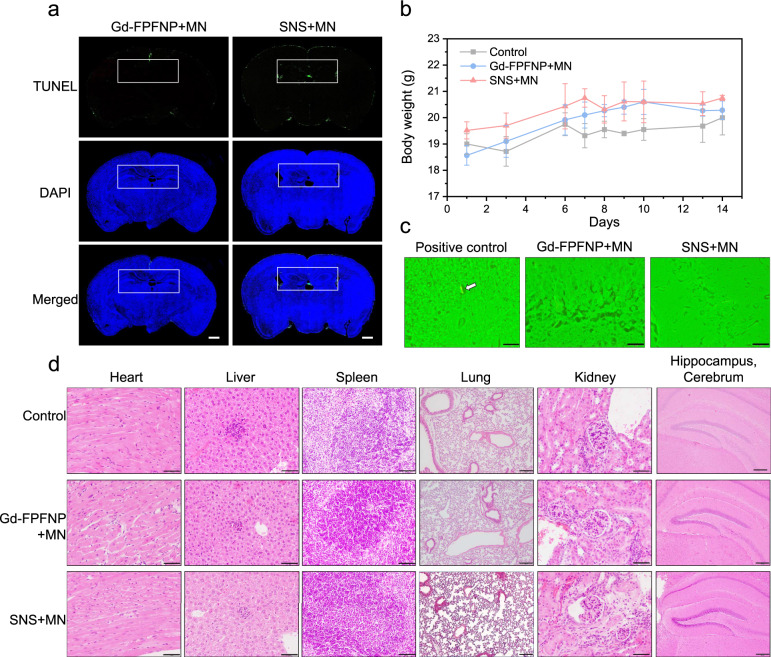
Safety assessment of stem cell–nanoparticle system (SNS) and Gd-Fu@IO@PVA/Fu nanoparticle (Gd-FPFNP). **a** TdT-mediated dUTP Nick-End Labeling (TUNEL) staining of the brains in healthy rats (*n* = 3) treated with Gd-FPFNP plus magnetic navigation (MN) or SNS plus MN at 21 days post intracarotid injection. Scale bars = 500 µm. The white rectangle indicates the site of the dentate gyrus (DG). The TUNEL images are representative of 3 images. **b** Body weight change of C57BL/6JNarl mice at 2 weeks post intracarotid injection with saline (control), Gd-FPFNP plus MN and SNS plus MN. The results were expressed as mean ± SD, *n* = 3 mice. **c** Fluoro-Jade C (FJC) staining of dentate gyrus area in brains of C57BL/6JNarl mice (*n* = 3) at 14 days after intracarotid injection with SNS + MN and Gd-FPFNP + MN (the white arrow indicates the neuropathic cells in positive control group). The FJC staining images are representative of 6 images. **d** H&E staining of major organs (heart, liver, spleen, lung, kidney, and brain) in C57BL/6JNarl mice (*n* = 3) at 14 days post intracarotid injection with Gd-FPFNP plus MN and SNS plus MN. Scale bar = 50 µm (heart, liver, kidney, and spleen) and 200 µm (lung and hippocampus cerebrum). The H&E staining images are representative of at least 6 images.

To understand the local and systemic safety profile of Gd-FPFNP and SNS, we performed safety assessments in healthy rats and mice. First, as noted in Fig. 4b, the concentration of Gd in the organs would be less than 10 ppb at 7 days post administration of SNS, indicating that the Gd would not permanently remain in vivo and cause toxicity. At 21 days post treatment with Gd-FPFNP plus MN or SNS plus MN, negligible background apoptosis in the dentate gyrus (DG) was detected in the brain of rats by TUNEL assay (Fig. 6a) indicating that both formulations were locally biocompatible in brains.

Furthermore, we performed 14-day systemic and local toxicity tests using C57B/6 mice to evaluate the safety of Gd-FPFNP and SNS. Over the 14 days, the body weight of all mice showed a similar tendency without body weight loss (Fig. 6b), and all clinical observations were within normal range. To evaluate the potential neuropathy that might be induced by the deposition of Gadolinium, Fluoro-Jade C (FJC) staining of the dentate gyrus area was performed in mice treated with Gd-FPFNP or SNS. Encouragingly, we did not detect any degenerating neurons in the area (Fig. 6c) compared with a positively labeled sample. The histological analyses of vital organs including heart, liver, spleen, lung, kidney, and brain at 14 days after intracarotid injection revealed that all tissues were within normal range and free of any abnormality related to gadodiamide or UMSC such as neuron degeneration, inflammatory, or lesions (Fig. 6d).

Fucoidan has been demonstrated to possess anti-inflammatory and neuroprotective functions^52,53^. Correspondingly, we observed that Gd-FPFNP with the fucoidan coating demonstrated a potential protective effect after NCT treatment. As shown in Fig. S22, serum levels of pro-inflammatory factors including IL-1β, IFN-γ, TNF-α, IL-12 and MCP-1 decreased and those of anti-inflammatory cytokines such as IL-10 and G-CSF increased after treatment with Gd-FPFNP and Gd-FPFNP plus NCT. These results suggest that Gd-FPFNP might provide a protective effect and faster recovery for the brain tissue after Gd-NCT.

## Discussion

In this study, we integrated nanotechnology-derived Gd-FPFNP with UMSCs to develop a stem cell–nanoparticle system (SNS) possessing anti-inflammatory and neuroprotective activities as a cell-based vehicle for augmenting the therapeutic index of Gd-NCT. Due to the drug-concealing properties of Gd-FPFNP, the UMSCs in SNS had a higher content of Gd agent compared to the free gadodiamide group (Fig. 2g). After internalization of Gd-FPFNP, the SNS could be magnetically guided toward the GBM to enhance the biodistribution. Moreover, the SNS could only sustain its biological stem cell functionality when the gadodiamide was encapsulated in the Gd-FPFNP, that effectively prevent the release of cytotoxic Gd ions.

Our results demonstrated that the SNS had the capability to protect the gadodiamide from degradation and disassociation (Fig. 2e), improve its biodistribution (Fig. 4b, c), and specifically deliver it into GBM to significantly increase the T/B and T/N ratios (Fig. 4d, e). The T/B and T/N ratios are important factors when developing a NCT agent. Taking B-NCT as an example, as boron-based agents show poor accumulation and non-specificity toward tumor, a long administration time of up to several hours is needed in the clinical application to achieve an adequate boron concentration at the tumor and the proper T/B ratio^15–17^. However, the irradiation time of B-NCT in the clinical trial was approximately 15 to 40 min, which was limited by OAR (i.e., the maximum tolerated dose of adjacent normal tissue)^54,55^. In contrast, as our SNS platform showed tumor specificity (Figs. 3c and 4b, c) and yielded a high T/B and T/N ratio (Fig. 4d, e), the exposure time can be extended (1 h 36 min) without inducing damage to the adjacent tissues (Fig. 5d).

The major photoelectric products produced by gadodiamide are gamma radiation, internal convergent (IC) electrons, and Auger electrons. During neutron irradiation, Auger electrons would be weakened by cell delivery due to their short travel distance but tumor inhibitory effects of Gd-NCT can still be achieved due to the gamma rays and IC electrons emitted from Gd. These electron emissions have longer traveling distances and can cover the whole tumor area with sufficient energy deposition to cause DNA damage, thus overcoming the limitation of short-range coverage in B-NCT^9,11^.

However, the longer traveling distances might also cause safety concerns when the Gd agents are not localized in the tumor. The TUNEL assay demonstrated that although Gd-FPFNP was able to penetrate the leaky BBB, its random distribution in the brain (Fig. S14 and Fig. 4c) would induce damage throughout the whole-brain area (Fig. 5d). In contrast, SNS was capable of homing to GBM and specifically localized in the tumor area to effectively eliminate the tumor without damaging the adjacent normal tissues. Therefore, we consider the SNS with tumor-homing ability to be a superior NCT agent delivery system compared with nanoparticles for the treatment of GBM using NCT.

The enhancement of tumor localization would be reflected in the therapeutic efficacy of Gd-NCT. When administered via intracarotid injection in the orthotopic GBM rat model, SNS with application of MN reduced the tumor size and extended median survival more than 2.5-fold compared with the control group (Fig. 5). Besides, SNS could improve the therapeutic window in clinical application, which is related to the properties of drugs in the circulation and in particular their pharmacokinetics, biodistribution, tumor localization ability, and safety/toxicity.

Importantly, as shown in Table S3, the dose of Gd used in SNS (0.2 mg kg^−1^ in rats) was substantially lower than that of Gd-containing nanomedicines previously used in NCT treatment (1.15–94.3 mg kg^−1^ in rats). For instance, Hofmann and Peng et al. reported the use of Gadobutrol and Gd-DTPA for Gd-NCT at doses of 18.14 mg and 206.25 μg respectively in mice, which were approximately 500- and 5.75-fold higher than the dose used in our study^10,56^. The reduced dose did not compromise the therapeutic efficacy (Fig. 5), but largely enhanced the safety profile of SNS. For instance, low-dose Gd may prevent the development of nephrogenic systemic fibrosis^13,57^. The reduction of pro-inflammatory factors and enhancement of anti-inflammatory cytokines in serum of the mice treated with Gd-FPFNP demonstrated that Gd-FPFNP comprising fucoidan can trigger potent anti-inflammatory and neuroprotective effects. The in vivo results indicated that Gd-FPFNP could be beneficial for recovery of brain function after Gd-NCT therapy (Fig. S22).

In summary, SNS has been demonstrated to overcome the current limitations of Gd agents, including off-target effects and rapid metabolism, that hamper the efficiency of Gd-NCT. Moreover, SNS achieved significant tumor inhibition and extended survival effects with only one NCT radiation treatment under an ultra-low dose of Gd agent. Our results indicate that SNS is a powerful potential strategy for Gd-NCT of GBM and its extended application to a wider variety of tumor types is worth exploring.

## Methods

### Ethics statement

The animal use and experimental protocols were approved by Institutional Committee of Animal Research of China Medical University under IACUC number CMU 2020-014. The collected human umbilical cord tissues approved by the Institutional Review Board (IRB) of the China Medical University Hospital, Taiwan under IRB number CMUH-110-REC-1-068.

### Materials

Fucoidan (from fucus vesiculosus), sodium meta-periodate, chloroform, 1,2-hexadecanediol (97%), Fe (acac)_3_, oleic acid (90%), olecylamine (>70%), sodium azide, and benzyl ether (99%) were purchased from Sigma Aldrich. Polyvinyl alcohol (PVA, Mw: 25k) was purchased from Fluka Chemical Co.. Quantum dots (QDs, CdSe/ ZnS with Excitation/Emission = 612/620 nm) was purchased from Ocean nanotechnology. Cy5.5 was purchased from Life Science Technology.

The antibodies used in the study are described as below: the antibodies used in identifying the stemness of MSC including anti-CD13 (clone WM15, Cat No: 555394), anti-CD29 (clone MAR4, Cat No: 555443), anti-CD44 (clone G44-26, Cat No: 550988), anti-CD73 (clone AD2, Cat No: 550257), anti-CD90 (clone 5E10, Cat No: 559869), anti-CD105 (clone 266, Cat No: 560839, anti-CD166 (clone 3A6, Cat No: 559263), anti-CD49b (clone 12F1, Cat No: 555669), anti-CD1d (clone CD1d42, Cat No:563505), anti-CD3 (clone SK7, 340542), anti-CD10 (clone HI10a, Cat No: 561002), anti-CD14 (clone M5E2, cat No: 561712), anti-CD31 (clone MEC13.3, cat No: 550274), anti-CD34 (clone 581, cat No: 555824), anti-CD45 (clone HI30, 560975), anti-CD49d (clone 9F10, cat No: 560972), anti-CD56 (clone B159, cat No: 555518), anti-CD117 (clone 104D2, cat No: 340529), anti-HLA-ABC (clone G46-2.6, cat No: 555555), and anti-HLA-DR (clone G46-6, cat No: 560944) were purchased from BD Biosciences and were all diluted at 1:100. For the antibodies used in immunocytochemical assessment including the ones against luciferase (clone21, diluted at 1:200), GFAP (clone GA5, diluted at 1:1000), Tuj-1 (clone TU-20, diluted at 1:1000), or MAP-2 (clone AP20, diluted at 1:300) were purchased from Millipore. Anti-β-Actin (clone mAbcam 8226, diluted at 1:1000) was purchased from Abcam.

### Synthesis of hydrophobic superparamagnetic iron oxide nanoparticles (IO)

The superparamagnetic iron oxide nanoparticles (IO) were prepared following the protocol of Sun *et al*^58^. In brief, Fe(acac)_3_ (2 mmol), 1,2-hexadecanediol (10 mmol), oleic acid (6 mmol), and olecylamine (6 mmol) were added to a three-necked bottle and then mixed with benzyl ether (20 ml) and refluxed at 100 °C for 30 min under a nitrogen atmosphere. The solution was heated to 200 °C for 1 h and then to 285 °C for 30 min to complete the nucleation and growth of SPIOs. After cooling to room temperature, the product was collected by centrifugation at 6200 × *g* for 10 min and purified with ethanol three times.

### Synthesis of Gadodiamide-encapsulated FFNP, PPNP and FPFNP

Gd-FFNP, Gd-PPNP, and Gd-FPFNP were synthesized through a double emulsion process. For Gd-FFNP and Gd-FPFNP, the first aqueous water (0.2 ml) contains gadodiamide (30 mg) and fucoidan (2 mg); while for PPNP, it contains gadodiamide (30 mg) and PVA (2 mg). The first aqueous water was then added to the organic phase (4 mg IO in 0.4 ml chloroform) and emulsified by the pulsed ultrasound sonification using a homogenizer (Double Eagle Enterprise Co, Ltd, New Taipei City, Taiwan) to form the water-in-oil (W/O) solution.

Next, the second water phase (3 ml) containing fucoidan (30 mg), PVA (30 mg), or fucoidan (15 mg) plus PVA (15 mg) for Gd-FFNP, Gd-PPNP, and Gd-FPFNP, respectively, was emulsified with the W/O solution at 120 W for 60 sec to obtain the W/O/W emulsion. The chloroform was removed by an evaporator and the nanoparticles were purified using a magnetic selection equipment (MagniSort®, eBioscience). Finally, the nanoparticles were resuspended with distilled deionized water. To observe the behavior of cell association, quantum dots (QD, 0.5 mg ml^−1^) was mixed with IO in the hydrophobic phase during synthesis to facilitate fluorescent microscopy analysis.

### Characterization

The morphology of FFNP, FPFNP, and PPNP was observed using scanning electron microscopy (SEM) and transmission electron microscopy (TEM). The particle size and zeta potential of FFNP, FPFNP, and PPNP were analyzed by DLS (BECKMAN COULTER Delsa Nano C particle analyzer).

### Loading capacity, encapsulation efficiency, and in vitro drug release

The loading capacity (LC), encapsulation efficiency (EE) and release behavior of gadodiamide from Gd-FFNP, Gd-PPNP and Gd-FPFNP were examined using inductively coupled plasma mass spectrometry (ICP-MS). The nanoparticles were lyophilized, weighed, and quantified. The LC of the gadodiamide were calculated as follows:

1 $${{{{{\rm{LC}}}}}}\%=\frac{{A}}{{B}}\times 100\%$$

where *A* is the mass of encapsulated gadodiamide and *B* is the mass of Gd-FFNP, Gd-PPNP or Gd-FPFNP, while the EE was calculated as:

2 $${{{{{\rm{EE}}}}}}\%=\frac{{C}}{{D}}\times 100\%$$

where *C* is encapsulated drug and *D* is the total amount of drug used in the synthesis.

To monitor the release behavior of gadodiamide, Gd-FFNP, Gd-PPNP and Gd-FPFNP were placed in a dialysis tube with cellulose membrane (molecular weight cutoff = 1 kDa). The dialysis tubes were immersed in phosphate-buffered saline (PBS, 10 mL) at 37 °C with gentle shaking, and the aliquots of the PBS were collected at predetermined time points for quantification. The amounts of released gadodiamide were quantified using HPLC (Agilent Technologies 1200 series, San Diego, USA) with a C18 LiChrospher® column (250 × 4 mm, 5 μm, Merck, Darmstadt, Germany). The detector was operated at a wavelength of 210 nm, and the sample was eluted using an aqueous mobile phase containing triethylamine (15 mmol L^−1^) and glacial acetic acid (5 mmol L^−1^, pH 6.5–7) at a flow rate of 1.0 ml min^−1^ with ambient temperature^59^. The retention time of gadodiamide was at 4.5 min. All experiments were performed in triplicate.

### In vitro Gd^3+^ release

To monitor the Gd^3+^ dissociation behavior, free Gadodiamide and Gd-FPFNP were incubated in DMEM and added to a dialysis tube with cellulose membrane (molecular weight cutoff = 1 kDa). The dialysis tubes were immersed in phosphate-buffered saline (PBS, 10 mL) at 37 °C with gentle shaking, and the aliquots of the incubation medium were collected from 3 h to 24 h. The amount of Gd^3+^ was measured by addition of Xylenol orange agent and analyzed by a UV–vis detector^32^.

### Cells

The glioblastoma multiforme (GBM) cells including F98^60,61^, and GBM8401^62^ were selected to establish the GBM rat model and were purchased from American Type Culture Collection (ATCC® CRL-2397™) and Bioresource Collection and Research Center, Taiwan (BCRC Number: 60163), respectively. 4T1 cell was purchased from American Type Culture Collection (ATCC® CRL-2539™). All the cell lines used in the study were authenticated by the supplier using morphology, karyotyping, or PCR-based approaches

Luciferase-expressing F98 (F98-Luc) cells were obtained by encoding F98 with the luciferase gene, respectively. Luciferase cDNA from plasmid luciferase-pcDNA3 (#18964, Addene) were transferred into pIRES (Clontech) by specific restriction enzyme linker (EcoR1 and Nhe1) to build as the construct of pSF-luciferase. The PiggyBac vector pPB-CMV-MCS-EF1α-Puro, which contains the multiple cloning sites (MCS), PiggyBac terminal repeats (PB-TRs), core insulators (CIs) and puromycin selection maker driven by the human EF1α, were used as the base vector (System Bioscience). DNA fragment containing luciferase (from pSF-luciferase) was PCR amplified and sub-cloned into the pPB-CMV-MCS-EF1α-Puro vector, in front of the coding region of EF1α (abbreviated pPB-luciferase). To generate F98-Luc stable cells, the above pPB-luciferase plasmid was co-transfected with a PiggyBac transposase (System Biosciences) into F98 cells by Amaxa NucleofectorTM 2b device (Lonza), and stable cells were selected by puromycin.

UMSCs were collected from human umbilical cord tissues, and the process was approved by the Institutional Review Board (IRB) of the China Medical University Hospital (Taichung, Taiwan). Throughout the whole study, we did not detect mycoplasma contamination of the cells via Hoechst DNA stain method and agar culture method.

### Glioma cell culture

F98, F98-Luc and GBM8401 cells were cultured in Dulbecco’s modified Eagle’s medium (DMEM, Gibco) with fetal bovine serum (10 % v/v, Gibco) and penicillin-streptomycin solution (0.5% w/v, Gibco), at 37 °C under 5% CO_2_ and 95% air (both v/v) for further use.

### Preparation, isolation, and characterization of UMSCs

To collect UMSC from human umbilical cord, the tissues were washed three times with Ca^2+^- and Mg^2+^-free PBS (DPBS, Life Technology) and mechanically cut using scissors in a midline direction. The vessels of the umbilical artery, vein, and outlining membrane were dissociated from the Wharton’s jelly (WJ). The jelly content was extensively cut into pieces with the size smaller than 0.5 cm^3^, treated with collagenase type 1 (Sigma, St Louis, USA), and incubated at 37 °C in a 95% air/5% CO_2_ humidified atmosphere for 3 h. The explants were then cultured in DMEM containing 10% fetal calf serum (FCS, Gibco) and antibiotics at 37 °C in a 95% air/5% CO_2_ humidified atmosphere. The cells were left undisturbed for 5–7 days to allow the migration of the cells from the explants.

The cellular morphology of UMSCs would become homogenously spindle shaped after 4–8 passages (as shown in Fig. S5a), and the specific surface molecules of the UMSC were characterized by flow cytometry.

### Flow cytometry

To analyze the surface expression of UMSC, the cells were detached with 2 mM EDTA in PBS, washed with PBS containing BSA (2 %) and sodium azide (0.1 %) and incubated with the respective antibody conjugated with fluorescein isothiocyanate (FITC) or phycoerythrin (PE). As a control, cells were stained with mouse IgG1 isotype-control antibodies. The antibodies to CD1q, CD3, CD10, CD14, CD31, CD34, CD45, CD49d, CD56, CD117, HLA-DR, CD13, CD29, CD44, CD49b, CD73, CD90, CD105, CD166 and HLA-ABC were purchased from BD Biosciences. Cells were analyzed using a FACScan with CellQuest Analysis (BD Biosciences) and FlowJo software v9.3.2 (TreeStar Inc.). The gating strategy was provided in Fig. S5. Results are expressed as the percentage of positively stained cells relative to total cell number. For quantitative comparison of surface protein expression, the fluorescence intensity of each sample was presented as median fluorescence intensity (MFI).

### Labeling of UMSCs with Gd-FPFNP and the quantification of intracellular IO/ Gd

To construct the SNS, UMSCs (1 × 10^5^) were seeded in 6-well plates and cultured with serum-free medium containing Gd-FPFNP at various iron concentrations (0, 1, 10, 50, 100, 150 μg Fe ml^−1^) for 6 h with a magnet applied under the culture plate. After incubation for different times (2 h, 6 h, 12 h, 24 h and 5d), the medium was removed, and the cells were washed with PBS (pH 7.4) for three times. It is noted washing the cells for three times is a critical step to ensure the complete removal of Gd-FPFNPs and free-form Gd in the medium. The fluorescent dye of quantum dots (Qd, Sigma) incorporated in Qd-labeled FPFNP (Qd-FPFNP) served as a marker for quantitative determination of the cellular uptake of Gd-FPFNP using flow cytometry (FACSCalibur, BD Bioscience) and CLSM (Carl Zeiss LSM510 l).

The subcellular location of Gd-FPFNP in UMSC was determined by TEM. In brief, SNS (at 100 μg Fe ml^−1^) was fixed with glutaraldehyde (2.5%) in sodium cacodylate (0.05 M, pH 7.4) for 40 min and embedded in agarose (2%). The embedded cells were then stained with osmium tetroxide (2%) and uranyl acetate (0.5%) and processed for ultrathin sectioning. Images were taken using TEM (Philip CM-120, Eindhoven, Netherlands) at an acceleration voltage of 80 kV.

The viability of SNS was assessed by MTT assay according to the manufacturer’s procedures (Sigma). UMSCs were incubated with Gd-FPFNP at various concentrations (0, 1, 10, 50, 100, 150 μg Fe ml^−1^) for 2 h and the culture medium was replaced with medium containing MTT (0.8 mg ml^−1^). After further incubation for 4 h, the medium was examined at absorbance of 560 nm using a 96-well microplate reader (Spectra max190-Molecular Devices).

### Proliferation, migration and differentiation assays of UMSC and SNS

To examine cellular proliferation and migration, bromodeoxyuridine (BrdU) incorporation and transwell migration assays were performed with/without magnetic navigation. Proliferation of SNS or UMSCs with/without application of the magnet (5 mm in diameter, 0.5 mm in height, 1000G) was tested as indicated using BrdU chemiluminescence immunoassay kits (10 μM, Roche) and further confirmed by counting Trypan blue stained cells.

After starvation (incubation in the medium lacking supplements) for 4–6 h, UMSCs were incubated in medium for 2 days and pulse loaded with BrdU (10 μM) for 12 h. The cells were then incubated with anti-BrdU-peroxidase for 90 min and staining was developed by incubation with substrate solution for 3 min. Plates were read with Lmax microplate luminometer (Molecular Devices). Results were analyzed and presented as percent (%) increase over control.

Cell migration was assessed as described previously with modifications^63^. In brief, SNS or UMSCs were placed in the upper chamber (transwell: 6.5-mm diameter, 5.0-mm pore size) according to the manufacturer’s instructions (Costar, #3421). SDF-1α (100 ng/ml, R&D System, positive control) was added to the lower chambers. The assays were conducted over a 4-h incubation period at 37 °C in a 5% CO_2_ incubator. Because most cells stay on the lower side of the membrane after migration, quantification can be performed by simply counting the adhered cells on the lower side of the membrane under microscopy.

Adipogenic differentiation was induced according to the method described below. In brief, confluent monolayer cultures of SNS or UMSCs were grown in adipogenic differentiation medium consisting of DMEM-high glucose (DMEM-HG, Sigma), penicillin (100 U ml^−1^), streptomycin (100 mg ml^−1^), insulin (100 mM), 3-isobutyl-1-methylxanthine (500 mM), dexamethasone (1 mM), indomethacin (100 mM) and FCS (10%). Cells maintained in regular UMSC medium served as a negative control. The adipogenic differentiation medium was changed three times per week. To assess adipogenic differentiation, cells were stained with oil red O (0.3%) for 10 min at room temperature, and counterstained with hematoxylin.

To induce osteogenic differentiation, confluent monolayer SNS or UMSC cultures were grown in DMEM-high glucose (DMEM-HG, Sigma) containing penicillin (100 U ml^−1^), streptomycin (100 mg ml^−1^), L-ascorbic acid 2-phosphate (50 mg ml^−1^), b-glycerophosphate (10 mM), dexamethasone (100 nM), and FCS (10%). Cells maintained in regular UMSC medium served as negative controls. The osteogenic differentiation medium was changed three times per week. Levels of osteogenesis were determined using Alizarin red S staining (1 %) to detect calcium mineralization. Chondrogenic differentiation of SNS or UMSCs was induced using a high-density pellet cell culture system. Cells were washed in serum-free chondrogenic differentiation medium consisting of DMEM-HG, penicillin (100 U ml^−1^), streptomycin (100 mg ml^−1^), l-ascorbic acid 2-phosphate (50 mg ml^−1^), proline (40 mg ml^−1^), sodium pyruvate (100 mg ml^−1^), dexamethasone (100 nM), and ITS-plus (10 mg ml^−1^ bovine insulin, 5.5 mg ml^−1^ transferrin, 5 mg ml^−1^ sodium selenite, 4.7 mg ml^−1^ linoleic acid, and 0.5 mg ml^−1^ bovine serum albumin). Aliquots of 250,000 cells were resuspended in chondrogenic differentiation medium, centrifuged at 250 × *g*, and TGF-ß1 (10 ng ml^−1^, R&D Systems) was added. Pellets maintained in chondrogenic differentiation medium without TGF-β1 served as negative controls. Medium was changed twice a week. Chondrogenic differentiation of pellet cultures was confirmed histologically using Alcian blue staining of sulfated proteoglycans.

To evaluate the ability to differentiate into vascular tube, SNS or UMSCs was cultured in EBM (Cambrex) on 24-well plates which were precoated with Matrigel (300 μL well^−1^; Becton Dickinson) and vascular endothelial growth factor (VEGF, 10 ng ml^−1^, Sigma) for 2–3d. To induce neural cell differentiation, SNS or UMSCs was incubated with DMEM using a three-step method. Briefly, in the neural induction step, cells were plated at low density on 6-well plates containing fibronectin and exposed sequentially to: (1) DMEM-HG (Sigma) containing hUCS (2 %) or FCS (10 %) and bFGF (10 ng ml^−1^, R&D System) for 24 h; (2, the neural commitment step) DMEM-HG containing β-mercaptoethanol (1 mM, βME, Sigma) and NT-3 (10 ng ml^−1^ R&D Systems) for 2 days; and (3, the neural differentiation step) DMEM-HG containing NT-3 (10 ng ml^−1^, R&D Systems), NGF (10 ng ml^−1^, R&D Systems), and BDNF (50 ng ml^−1^, R&D Systems) for 3 to 7 days. Following cell differentiation, the cells were subjected to immunohistochemical analysis.

### Observation of UMSC using MRI

The UMSC were seeded in 6-well culture plates and treated with gadodiamide or Gd-FPFNPs with various Gd concentrations (0.01, 0.1, 1 and 10 μg ml^−1^), followed by dispersing in agarose (2 %). Images were obtained using a 7-Tesla (7-T) PharmaScan (Bruker). T1-weighted images were obtained by T1-FLASH sequences with the parameters set as follows: TE = 9 ms, TR = 500 ms, matrix size = 256 × 256, and NEX = 16. T2-weighted images were obtained by T2 RARE sequences with the parameters set as follows: TE = 19 ms, TR = 3000 ms, matrix size = 256 × 256, and NEX = 1.

### Relaxometric measurement

Different concentration (0.1–5 mM) of free gadodiamide and Gd-FPFNP were prepared for the measurement of T1 (spin-lattice relaxation time) MR relaxation time, while FPFNP and Gd-FPFNP were prepared for the measurement of T2 (spin-spin relaxation time) MR relaxation time using a relaxometer (0.5 Tesla, 20 MHz, 37 °C). Relaxation rates (1/T1 and 1/T2) were measured and plotted against the concentrations of Gd or IO. The relaxivities of *r*_1_ and *r*_2_ were calculated from the slopes of curves.

### Immunocytochemical assessment

Cells were washed with PBS and fixed for 30 min at room temperature in 4% paraformaldehyde. The fixed cells were washed with PBS and treated for 30 min with blocking solution (10 g L^−1^ BSA, 0.03% Triton X-100, and 4% serum in PBS). Cells were incubated overnight at 4 °C with antibody against luciferase (1:200, Millipore), β-Actin (1:1000, Millipore), GFAP (1:1000, Millipore), Tuj-1 (1:1000, Millipore), or MAP-2 (1:300, Millipore) (labeled with FITC or Cy3 fluorochromes) and then rinsed 3 times in PBS on the immunofluorescence-labeled slides before subjecting to the Carl Zeiss LSM510 laser-scanning confocal microscope for the analysis.

### Co-culture of glioma cells with SNS

Human GBM8401 cells infected with pDsRed-N1(Clontech) and cultured in DMEM medium supplemented with fetal bovine serum (10%), penicillin (100 U ml^−1^), and streptomycin (100 μg ml^−1^) in a humidified atmosphere with 5% CO_2_ at 37 °C to stably express red fluorescent protein (RFP). UMSCs transfected with Lenti-eGFP for GFP expression (UMSC-GFP) were labeled with FPFNP (100 μM) to construct GFP-SNS. To investigate the interaction between GBM8401-RFP and GFP-SNS mimicking the in vivo environment, GBM8401-RFP was co-cultured with GFP-SNS (ratio, 10:1) for 7 days. The spontaneous cellular fusion phenomenon was first examined without a fusogenic agent, and then the fusion progeny was examined by colocalization imaging by CLSM (Carl Zeiss LSM510 l). Dual-color RFP^+^/GFP^+^ cells were examined using FACS.

### Animals

All the animals including the female F344/NNarl rat (RMRC21002) and the male C57BL/6JNarl mice (RMRC11005) performed in the studies were purchased from National Laboratory Animal Center, Taiwan. The animal study protocol was approved by Institutional Animal Care and Use Committee (IACUC) of China Medical University under IACUC number CMU 2020-014. The number of the performed animals were provided in the figure legends related to each experiment. All the animals were housed under the specific pathogen free (SPF) condition (i.e., temperature:23 ± 2 °C, humidity: 50 ± 10%, 12-h light/dark cycle) with free access to food and tap water.

The humane endpoints of the rats and mice were set based on their body condition. First, this included the loss of 20–25% body weight over a few days or gradually. Second, the loss of appetite or weakness including inability to rise or ambulate for 24–36 h. Third, abnormal rises in body temperature or blood chemical values. Fourth, loss of respiratory, circulation, digestive, urinary, musculoskeletal, nerve or skin system function. In some cases, this limit was exceeded on the last day of measurement and animals were euthanized as soon as possible. When the animals met the endpoints, humane euthanasia was performed by exposing the animals to carbon dioxide (CO_2_) until complete cessation of breathing and the absence of respiration are observed (usually 2 mins but a total of approximately 5–10 min may be required).

### Orthotopic GBM animal models

The rats (8 weeks) were either inoculated with F98 or F98 luciferase-expressing dells (F98-Luc) cells via the stereotaxic implantation^61^. In brief, the rats were anesthetized by intraperitoneal (i.p.) injection of chloral hydrate (Sigma-Aldrich). Anesthetized animals were placed in a stereotactic frame and the skull was drilled using a micro-burr. Intracerebral injection of tumor cells (1 × 10^5^ in 2 μl) into the right striatum was performed via a 26 gauge-Hamilton syringe (10 μl) with the following stereotaxic coordinates: AP = 1; *L* = 2.5; *V* = 4. After the completion of injection, the needle was held in the place for an additional 5 min. Bone wax was further used to seal the hole on the skull.

### GFP-SNS transplantation and in vivo MRI examination

After magnetic purification to remove the free form of Gd-FPFNP, UMSC-GFP labeled with Gd-FPFNP (GFP-SNS) were administrated to the F98-Luc-bearing rats (10 days post tumor inoculation) by different routes (intracarotid and intravenous) with various cell numbers (2 × 10^4^, 2 × 10^5^, 2 × 10^6^ cells). Immediately after the administration of UMSC, a magnet (5 mm in diameter, 0.5 mm in height, 0.5T) was stuck onto the skull of the MN groups to achieve enhanced localization of SNS.

MRI was performed on rats under anesthesia in a 3-Tesla (3-T) MRI scanner (GE) at various time points (12 h, 24 h, and 48 h) after cell administration. During scanning, rats were gently warmed on a thermostatically controlled heating pad. For spin-echo (SE) T1-weighted imaging (T1WI), the images were acquired with repetition time/echo time (TR/TE) of 500/15 ms, field of view (FOV) of 28 × 44 mm. Rapid Acquisition with Relaxation Enhancement (RARE) spin-echo sequence was used for fast T2-weighted imaging (T2WI, TE 50 ms, TR 3000 ms) with a 256 × 256 in-plane matrix and a 2.56 cm field of view (FOV). For each rat, we acquired 22 coronal and axial images with 0.7 mm thick slices.

### Cellular fusion between glioma and UMSC in vivo

At 2 days after GFP-SNS transplantation, the GBM were sampled and cut into two pieces. Half of the fresh tumor tissue was cut into pieces smaller than 0.5 cm^3^, treated with collagenase type 1 (Sigma) and incubated at 37 °C for 3 h to obtain the F98-Luc/UMSC-GFP mixed cellular explant. Intracellular staining of luciferase^+^ cells and GFP^+^ cells was examined using FACS to evaluate the percentage of dual-color fused cells. Next, the other half of the tumor sample frozen in liquid nitrogen was continuously sectioned at a thickness of 6 μm using a cryostat and observed for luciferase^+^/GFP^+^ double immunofluorescent cells under a fluorescence microscope.

### Quantitative RT-PCR (qRT-PCR)

Total RNA was extract from mice brains by the single-step isolation method using Trizol (Invitrogen) and then reverse transcribed with random hexanucleotides using the SuperScript III First-Strand Synthesis System (Invitrogen) as previously described^64^. PCR was performed with JumpStart Taq DNA polymerase (Sigma). Quantitative PCR (q-PCR) primers were synthesized by Invitrogen. For q-PCR, 50 ng of total RNA was typically used as template in 20 μl SYBR green PCR reactions that additionally contained 0.375 mM of each primer and 10 μl of SYBR green PCR mix (ABI) with 40 cycles of 15 s, 95 °C/60 s, 60 °C on an Applied Biosystems 7300. Relative gene expression levels were determined by the critical threshold (Ct) number and calculated using the 2^−ΔΔCt^ method, with GADPH used as the reference gene for all groups.

The primer sequences of SDF-1 and GAPDH were provided as following: SDF-1-rat-(F) CGCCAAGGTCGTCGCCG, SDF-1-rat-(R) TTGGCTCTGGCGATGTGGC, GAPDH-rat-(F) CCCATTCTTCCACCTTTGATGCT, GAPDH-rat-(R) CTGTTGCTGTAGCCATATTCAT.

### Quantification of Gd and Fe using inductively coupled plasma mass spectrometry

Quantification of the elemental gadolinium (Gd) and iron content in acid hydrolyzed tissue samples was performed by inductively coupled plasma mass (atomic emission) spectrometry (ICP-MS, Agilent 7800 ICP-MS system). The analytic parameter is described as follows:

Peripump/ISIS settings: At pre-run mode, uptake speed (nebulizer pump) 0.5 rps, uptake time 30 s, stabilize 20 sec. At post-run mode (probe rinse), rinse speed (nebulizer pump) 0.5 rps, rinse at rinse port (sample) 10 sec, rinse at rinse port (standard) 10 s.

Plasma settings: RF power 1550 W, RF matching 1.70 V, sample depth 10.0 mm, nebulizer gas 1.05 L/min, nebulizer pump 0.10 rps, S/C temperature 2 °C. Ion lenses condition: extract 1 0 V, extract 2 −200 V, omega bias −80 V, omega lens 7.6 V, cell entrance −40 V, cell exit −60 V, deflect 0.6 V, plate bias −55V. For Gd ions, the analytical range of this instrument has been set from 0.1 to 1000 ng ml^−1^.

To analyze exocytosis of Gd from SNS, Gd content in the medium was measured by ICP-MS at 1, 2, 6, and 14 days. For cell samples, the number of the collected cell was determined, and the cells were further digested with concentrated nitric acid (Fisher Scientific) at 80 °C for 60 min prior to ICP-MS analysis. For samples derived from the brain tissues, organs, or blood, a pre-processing including desiccation at 90 °C for 4–6 h, weighting, and digestion with concentrated nitric acid (Fisher Scientific) at 80 °C for 60 min was performed. The analytic results of tissues were compared with standard curve generated from internal standards (Geel, Belgium) and the National Institute of Standards and Technology (Gaithersburg, Md). The Gd content in tissues was expressed as percentage of injected dose per gram tissue (ID%/g).

### Immunohistochemical assessment

Animals were anesthetized with chloral hydrate (0.4 g kg^−1^, i.p. injection) and fixed by transcranial perfusion with saline followed by immersion in 4% paraformaldehyde. Tumor tissue samples were dehydrated in 30% sucrose, frozen on dry ice, and then cut in a series of adjacent 6-μm-thick coronal sections using a cryostat. Sections were stained with H&E and Prussian blue (for identifying the iron) for observation by light microscopy (Nikon, E600). Each section was immunostained with primary antibodies against luciferase (1:100) followed by secondary antibodies conjugated with FITC (1:500; Jackson Immunoresearch), and then analyzed using a Carl Zeiss LSM510 laser-scanning confocal microscope. The percentage of colocalized Luciferase^+^/GFP^+^ cells was semi-quantified as the ratio of double-positive cells to total cell number under 10 high-power fields using Image J (NIH).

### Semi-quantitative grading of iron in tumor tissue

Prussian blue was used to stain the Gd-FPFNP in tumor tissue according to a previously reported method^65,66^. The SNS with MN, Gd-FPFNP with MN, and Gd-FPFNP were chosen for the determination of the amount and distribution of iron. Slices of brain tumor tissue were fixed with 4% formaldehyde and embedded in paraffin. At least three different areas of the 80× magnification view in the brain tumor tissue section were chosen for evaluation. The selected images were divided into 3× 3 square grids for evaluation of the presence of Gd-FPFNP. The score was semiquantitatively graded for iron content according to the following criteria: 0 = no iron; 1 = minimal or very small amounts; 2 = slight and patchy; 3 = moderate and diffuse; 4 = strong, extensive, and diffuse content.

### Biodistribution studies

Animals treated with Gadodiamide, Gd-FPFNPs, SNS, or SNS + MN were sacrificed at 12 h, 24 h, 48 h, and 7d post administration, and selected tissues and organs of interest were collected, weighed, treated nitric acid, and subjected to ICP-MS analysis. The injected dose per gram (% ID/g) was calculated as:

3 $$\%\frac{{{{{{\rm{ID}}}}}}}{{{{{{\rm{g}}}}}}}=100\%\times\frac{\frac{{{{{{\rm{Dose}}}}}}\; {{{{{\rm{in}}}}}}\; {{{{{\rm{Tissue}}}}}}}{{{{{{\rm{Totally}}}}}}\; {{{{{\rm{Dose}}}}}}\; {{{{{\rm{Injected}}}}}}}}{{{{{{\rm{Tissue}}}}}}\; {{{{{\rm{weight}}}}}}}$$

### Calculation of T/B and T/N ratios

Tumor-to-blood (T/B) and tumor-to-normal tissue (T/N) ratio were calculated as follows equations:

4 $${{{{{\rm{T}}}}}}/{{{{{\rm{B}}}}}}\; {{{{{\rm{ratio}}}}}}=\frac{{{{{{\rm{Gd}}}}}}\; {{{{{\rm{concentration}}}}}}\; {{{{{\rm{in}}}}}}\; {{{{{\rm{whole}}}}}}\; {{{{{\rm{brain}}}}}}}{{{{{{\rm{Gd}}}}}}\; {{{{{\rm{concentration}}}}}}\; {{{{{\rm{in}}}}}}\; {{{{{\rm{blood}}}}}}}$$

5 $${{{{{\rm{T}}}}}}/{{{{{\rm{N}}}}}}\; {{{{{\rm{ratio}}}}}}=\frac{{{{{{\rm{Gd}}}}}}\; {{{{{\rm{concentration}}}}}}\; {{{{{\rm{in}}}}}}\; {{{{{\rm{right}}}}}}\; {{{{{\rm{hemisphere}}}}}}\; {{{{{\rm{of}}}}}}\; {{{{{\rm{the}}}}}}\; {{{{{\rm{brains}}}}}}}{{{{{{\rm{Gd}}}}}}\; {{{{{\rm{concentration}}}}}}\; {{{{{\rm{in}}}}}}\; {{{{{\rm{left}}}}}}\; {{{{{\rm{hemisphere}}}}}}\; {{{{{\rm{of}}}}}}\; {{{{{\rm{the}}}}}}\; {{{{{\rm{brains}}}}}}}$$

### Bioluminescent imaging (BLI)

Animals were imaged with the IVIS Imaging System 200 Series (Caliper) to record bioluminescent signal emitted from the F98-Luc at 1 day before and 14 days after tumor inoculation. Animals were anesthetized with isoflurane and D-luciferin (Caliper) were administrated via intraperitoneal injection at the dose of 270 mg kg^−1^. Imaging acquisition was performed at 15 min after luciferin administration. For BLI analysis, region of interest (ROI) encompassing the intracranial area with bioluminescent signal was determined using Living Image 3.0 software (Xenogen Corp.) and the total photon flux was recorded.

### Therapeutic efficacy of Gd-NCT and the survival study

To determine the therapeutic effect of SNS on the survival of orthotopic glioblastoma tumor-bearing rats in vivo after irradiation, F98-Luc cells (2 × 10^6^) were suspended in PBS (0.2 mL) and stereotactically injected into the right striatum of rats. At 7–10 days after xenograft injection, SNS + MN (2 × 10^6^ cells in 500 μL, *n* = 6), SNS (2 × 10^6^ in 500 μL, *n* = 6), Gd-FPFNP (10 mg kg^−1^ in 500 μL, *n* = 6), Omniscan® (Gadodiamide, n = 6), or an equal volume of PBS (500 μL, *n* = 6) were injected into the left carotid artery (i.e., five treatment groups). To perform MN, the magnet of 5 mm in diameter, 0.5 mm in height, and 0.5 T magnetic field (Tun-Hwa Electronic Material Co., Ltd) was placed on the external skull of a F344 rat on the top of the tumor-bearing right brain for 12 h.

At 24 h after the administration of SNS, the rats were locally irradiated with a thermal neutron beam for 1 h and 36 min at a rate of 2 × 10^13^ neutron/cm^2^ Tsing Hua Open Pool Reactor (THOR, National Tsing Hua University, Taiwan). The exit of the neutron beam was 4.5 cm away from the head of the rat as shown in Fig. S23. The body of rats was shielded with poly-(methyl-methacrylate) and polyethylene complex plastic plates from unnecessary radiation^67,68^.

MRI was used to measure the tumor size before NCT. The tumor volume was quantified by compiling tumor areas from all slice images that contained tumors. The tumor boundaries can be distinguished in T2-weighted RARE images and the volume can further be quantified using ImageJ as following equation:

6 $${V}={a}\times {b}$$

where a is tumor area and b is slice thickness^61^. At 21 day post NCT, the rats were sacrificed, while the brains were sectioned and subjected to H&E staining for accurate measurement of the tumor volume using the following equation:

7 $${V}=\frac{{a}\times {{b}}^{2}}{2}$$

where *a* and *b* are the major and minor axes of the tumor sections as measured by a caliper. A Kaplan–Meier survival analysis was performed and reported as median survival times with 95% confidence interval. The statistical difference between the three conditions was determined by log-rank analysis.

### TUNEL staining assay

Brain sections were obtained from rats injected with free gadodiamide, Gd-FPFNP, or SNS after NCT (21 days post injection) to evaluate the potential toxicity. Cellular apoptosis was assayed by immunohistochemistry using a commercial TUNEL staining kit (DeadEnd Fluorimetric TUNEL system; Promega).

### Safety assessments

Healthy 8-week-old C57BL/6 male mice were treated with Gd-FPFNP + MN (*n* = 3), SNS + MN (*n* = 3), or saline (control, *n* = 3), and were observed for 14 days. During the period, we performed the clinical observation including mortality, body weight, and clinical symptoms. After 14 days, the mice were sacrificed and were subjected to histopathological assessments. The hearts, livers, lungs, spleens, kidneys, and brains were harvested and were preserved in neutral buffered formalin (10%, NBF) at room temperature for 96 h. The microtome was used to cut the sample with multiple sections with 4–6 um μm in thickness. These sections were subjected to H&E stain and the histopathological assessment carefully reviewed by a pathologist.

### Measurement of cytokines

Serum levels of cytokines (IL-1α, IL-1β, IFN-g, MIP-1α, MIP-1β, TNF-α, MCP-1, IL-12 (p40), IL-12 (p70), IL-10, G-CSF, and KC) were measured by Bio-Plex cytokine assays (ELISA Kit), which required as little as 12.5 μl of serum sample. Before analysis, all thawed samples were centrifuged to remove debris. All assay measurements were performed at least three times.

### Statistics and reproducibility

Statistical analysis was performed using Prism 9.0. All in vivo and in vitro experiments were repeated at least three times (*n* = 3 rats, mice or cell samples cultured in independent space such as plate or disk). Error bars represent standard deviation (SD) of three or more independent experiments. Two-tailed Student’s *t* tests were used to evaluate significance of mean differences between the control and the treated groups. Differences between groups were evaluated by one-way ANOVA with the Tukey’s multiple comparisons test unless otherwise noted. Kaplan–Meier survival curve were reported as median survival times with 95% confidence interval. Statistical differences between conditions were determined by log-rank analysis. A *P* value <0.05 was considered significant. All experiments in Figs. 2a, b; 3a–c; 5a; d; 6a; c, d and Supplementary Figs. 2a–d; 5a; 6; 7a; 8a; 9c, d were repeated for at least three times, and the performed numbers were provided in the regarding figure legends.

### Reporting summary

Further information on research design is available in the Nature Portfolio Reporting Summary linked to this article.

## Supplementary information

Supplementary Information

Reporting Summary

## Data Availability

The authors declare that the data supporting the findings of this study are available within the article, source data, and its Supplementary Information. Source data are provided with this paper.

## Source data

Source Data
