# Supplementary material for: Stem cell–nanomedicine system as a theranostic bio-gadolinium agent for targeted neutron capture cancer therapy
Source: Nat Commun. 2023 Jan 18;14:285. doi: 10.1038/s41467-023-35935-0 (PMC9845336; doi:10.1038/s41467-023-35935-0)
Supplement: Supplementary file 1 — Supplementary Information [file 41467_2023_35935_MOESM1_ESM.pdf]

Supplementary Information

Table S1. Encapsulation efficiency (%) and loading capacity (%) of Gd in various Fu-based nanoparticle formulations.

	Loaded Gd content (mg)	Encapsulation Efficiency (%)	Loading capacity (%)
Gd-FFNP	13.70	48.93	38.14
Gd-PPNP	15.47	55.27	18.78
Gd-FPFNP	16.69	59.64	28.57

Table S2. Semi-quantitative characterization of the distribution of Gd-FPFNP at the tumors by Prussian blue staining for different treatment conditions.

	Gd-FPFNP	Gd-FPFNP+MN	SNS+MN
Score[a]	1	1-2	3

[a] Perls' Prussian blue stained samples were graded semiquantitatively as follows: 0= no iron; 1=minimal or small amount; 2=slight and patchy; 3=moderate and diffuse; 4=strong, extensive, and diffuse content.

Table S3. A brief literature overview of Gd-containing nanomedicines or Gd-compounds used in NCT treatment for different types of tumors. The injected dose in mice has been converted to the same unit in rat for comparison.[1]

Gd formulation	Model	Dose (Gd-compound)	Translated Gd dose in rat ($mg\ kg^{-1}$)	Ref
Free gadobutrol	Subcutaneous melanoma/ mice	18.14 mg (Gadobutrol) for 25 g mice	≈94.33	2
Gd-DTPA-loaded chitosan NPs	Subcutaneous melanoma/ mice	2.4 mg (Gd-DTPA) for 25 g mice	≈3.44	3
Gd-DTPA/CaP NPs	Subcutaneous C26 tumor/ mice	206.25 µg (Gd-DTPA) for 25 g mice	≈1.15	4
SNS	Orthotopic GBM/ rat	109.3 µg (Gadodiamide)	0.2	This work

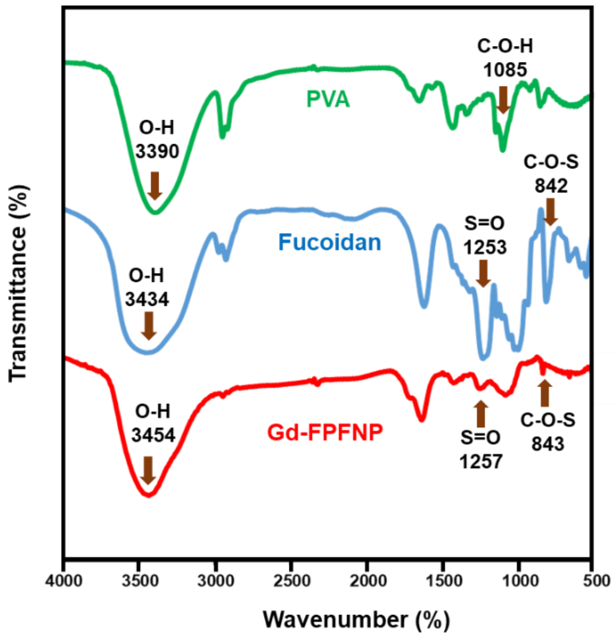

Figure S1. Fourier transform infrared (FTIR) spectra of PVA, fucoidan, and Gd-FPFNP.

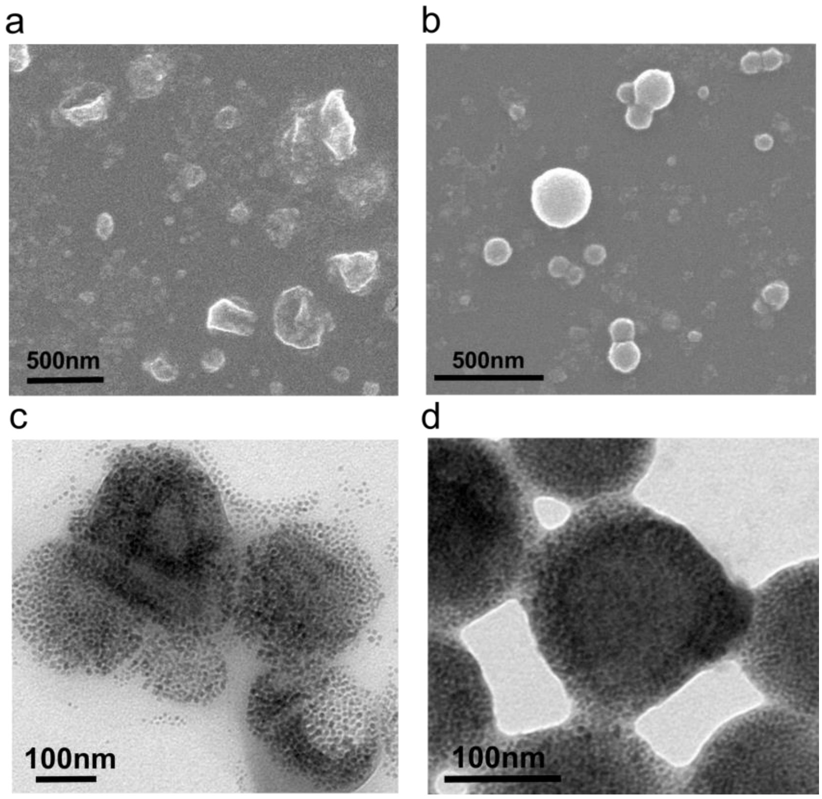

Figure S2. SEM image of a) Gd-FFNP and b) Gd-PPNP. TEM image of c) Gd-FFNP and d) Gd-PPNP. As fucoidan lacks amphipathic properties to stabilize the W/O interface, the Gd-FFNPs were not structurally stable and non-spherical nanostructures could be observed under electron microscopy (**Figure S2a, c**). In contrast, Gd-PPNP presented spherical and homogeneous structures (**Figure S2b** and **d**), attributed to the amphiphilic properties of PVA.

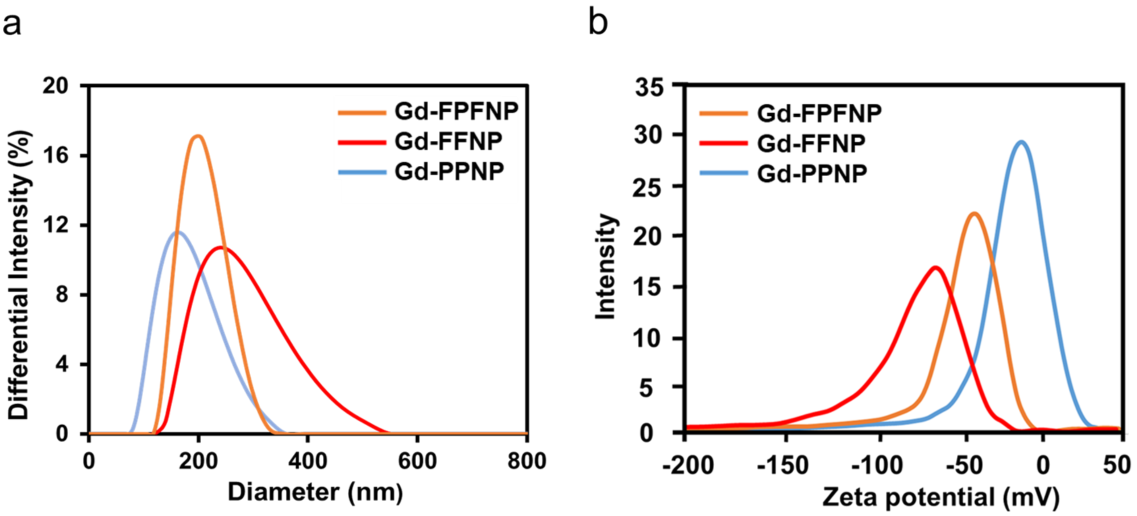

Figure S3. a) Size distributions of all nanoparticles measured using DLS. b) Zeta potentials of FFNP (red), FPFNP (orange), and PPNP (blue) were measured using DLS.

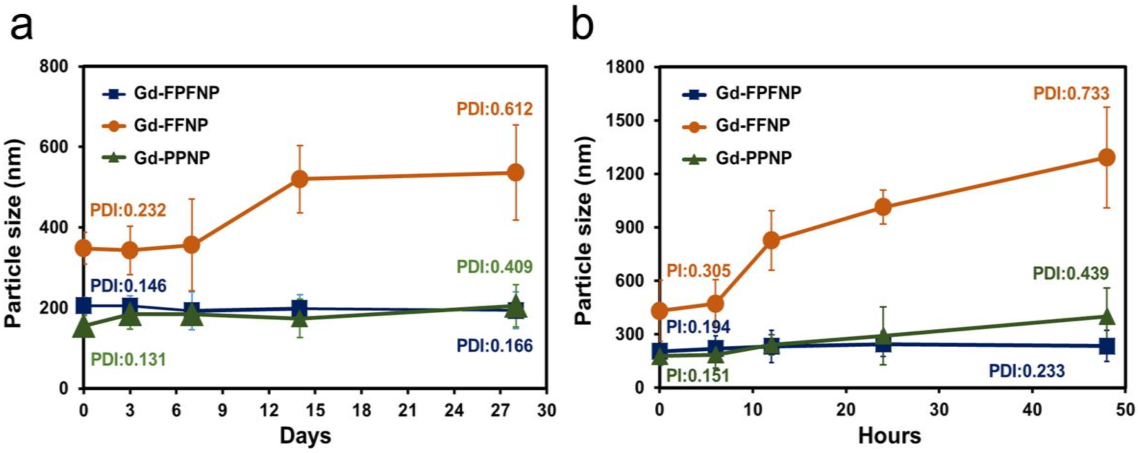

Figure S4. Stability of Gd-FPFNP, Gd-PPNP, and Gd-FFNP nanoparticles. The particles were suspended in a) PBS for 4 weeks or b) PBS with 10% fetal bovine serum (FBS) for 48 h. The results were expressed as mean ± SD, n = 3 independent nanoparticles. The size of Gd-FFNP substantially increased with time and became polydisperse at day 28 in both solutions. Although Gd-FPFNP and Gd-PPNP sustained colloidal stability in both solutions with minimal size change in the first 2 weeks, only Gd-FPFNP maintained a monodispersed condition at day 28 in both solutions.

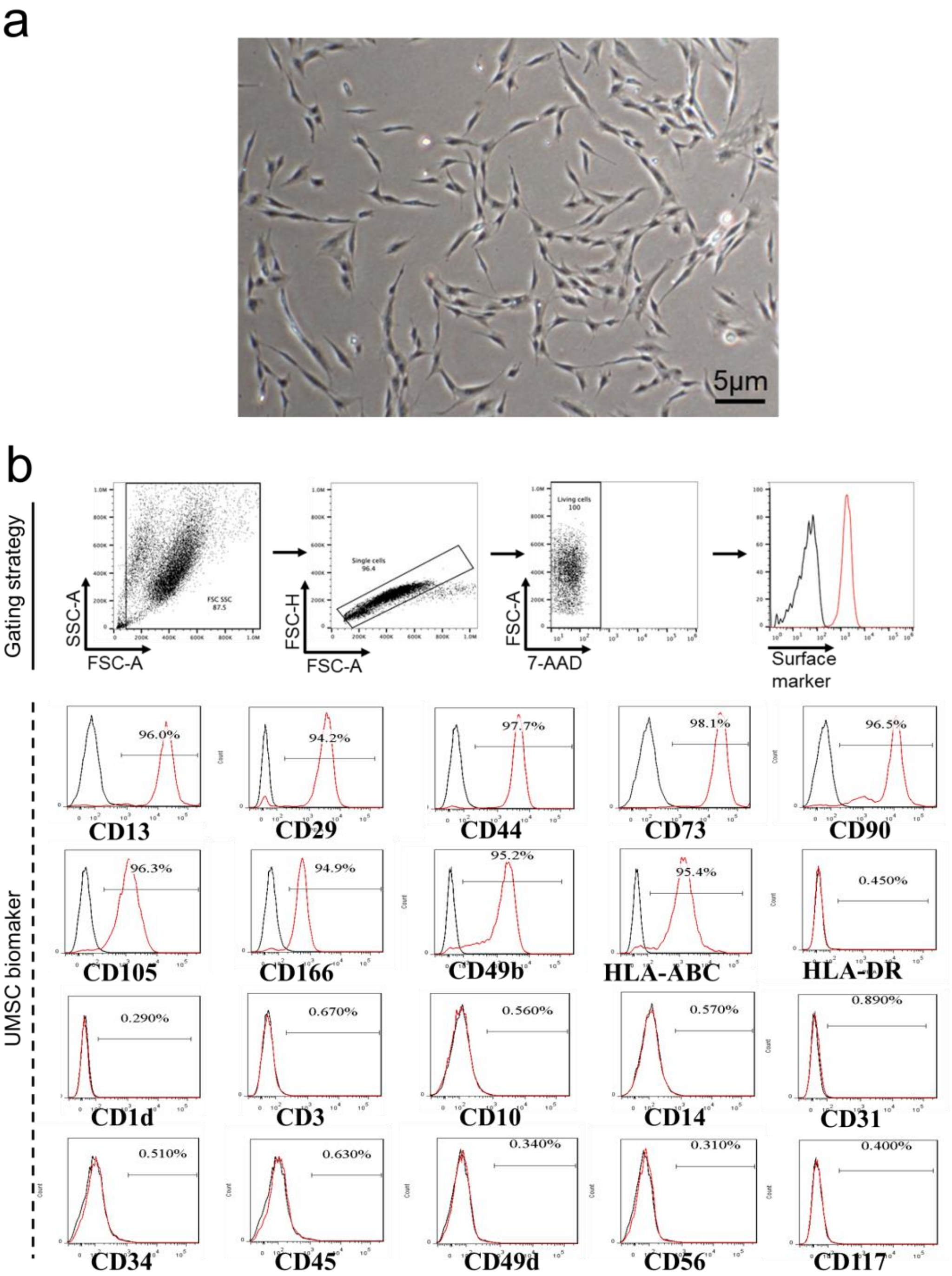

Figure S5. a) Cell morphology and b) biological properties of UMSCs. The UMSCs showed negative expression for CD1d, CD3, CD10, CD14, CD31, CD34, CD45, CD49d, CD56, CD117, and HLA-DR. In contrast, the surface markers of CD13, CD29, CD44, CD73, CD90, CD105, CD166, CD49b, and HLA-ABC were positive.

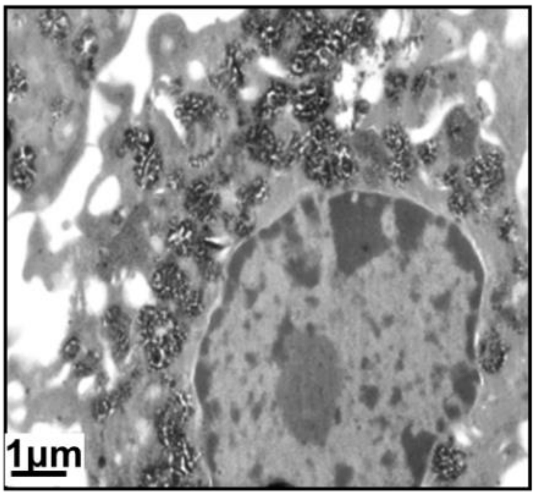

Figure S6. TEM image of Gd-FPFNP-treated UMSC (SNS).

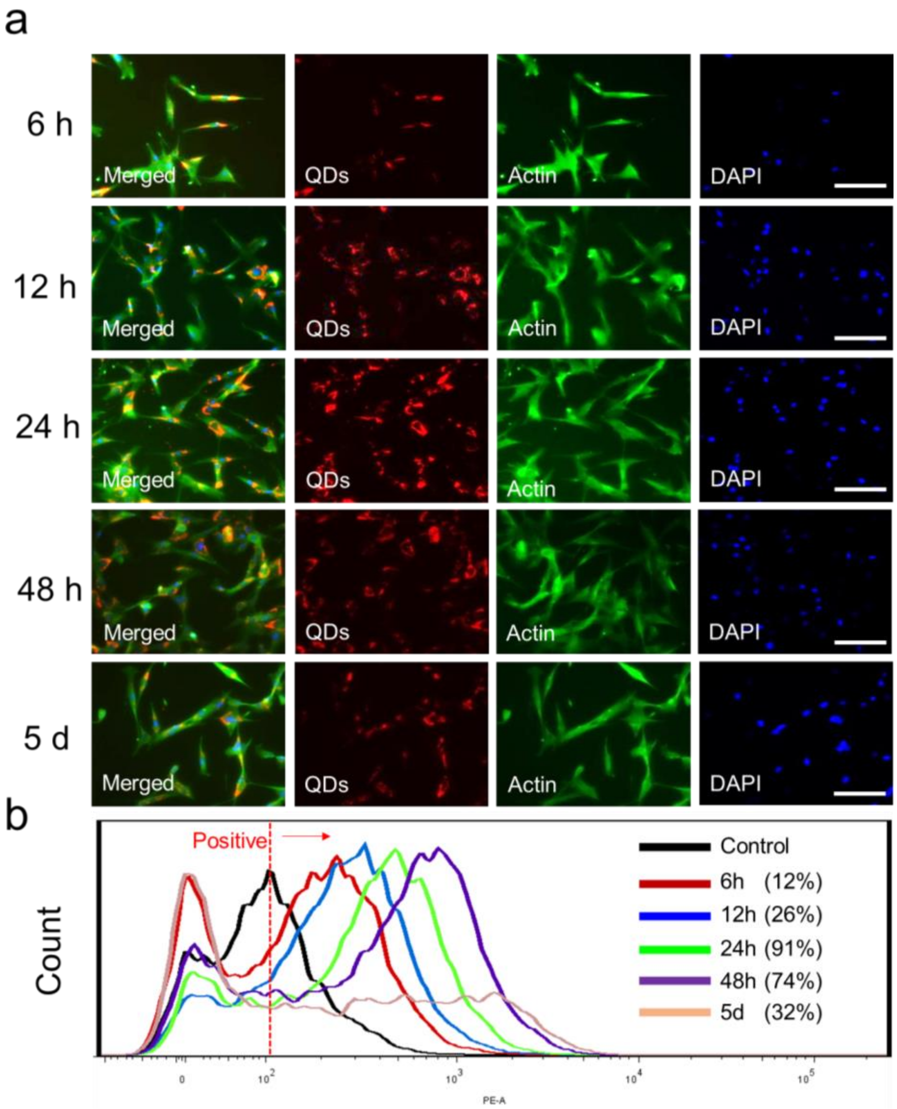

Figure S7. a) Cell uptake of UMSCs incubated with Gd-FPFNP for 6 h, 12 h, 24 h, 48 h, and 5 d, where the blue spots are the nuclei of the UMSC cells, the green fluorescence is cytoskeleton, and red indicates QD-labeled Gd-FPFNP. Scale bar = 20 μm. b) Flow cytometric analysis of cell uptake efficiency for UMSCs incubated with Gd-FPFNP for 6 h, 12 h, 24 h, 48 h, and 5 d.

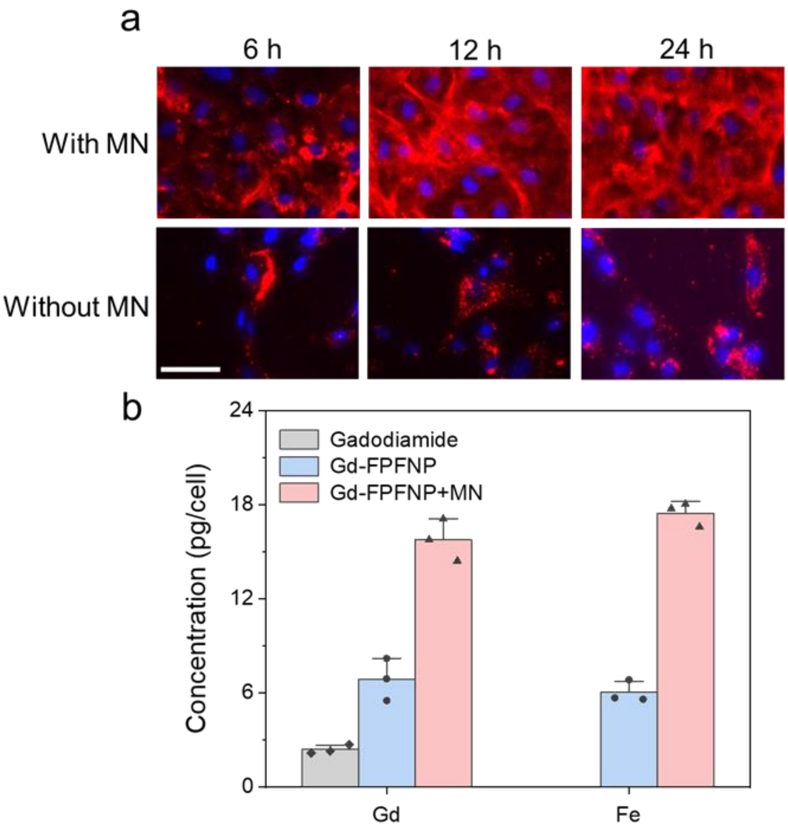

Figure S8. Cell uptake behavior. a) Uptake of UMSCs incubated with Gd-FPFNP with or without MN or 6 h, 12 h, and 24 h where the blue spots are the nuclei of the UMSC cells and red indicates QD-labeled Gd-FPFNP. Scale bar = 10 µm. *n* = 3 biologically independent UMSC samples with the images are representative of 3 images with similar results. b) Gd and Fe concentration of free gadodiamide and Gd-FPFNP after cell uptake for 12 h where free gadodiamide contains no Fe. Data were analyzed by ICP-MS. The results were expressed as mean ± SD, *n* = 3 biologically independent UMSC samples.

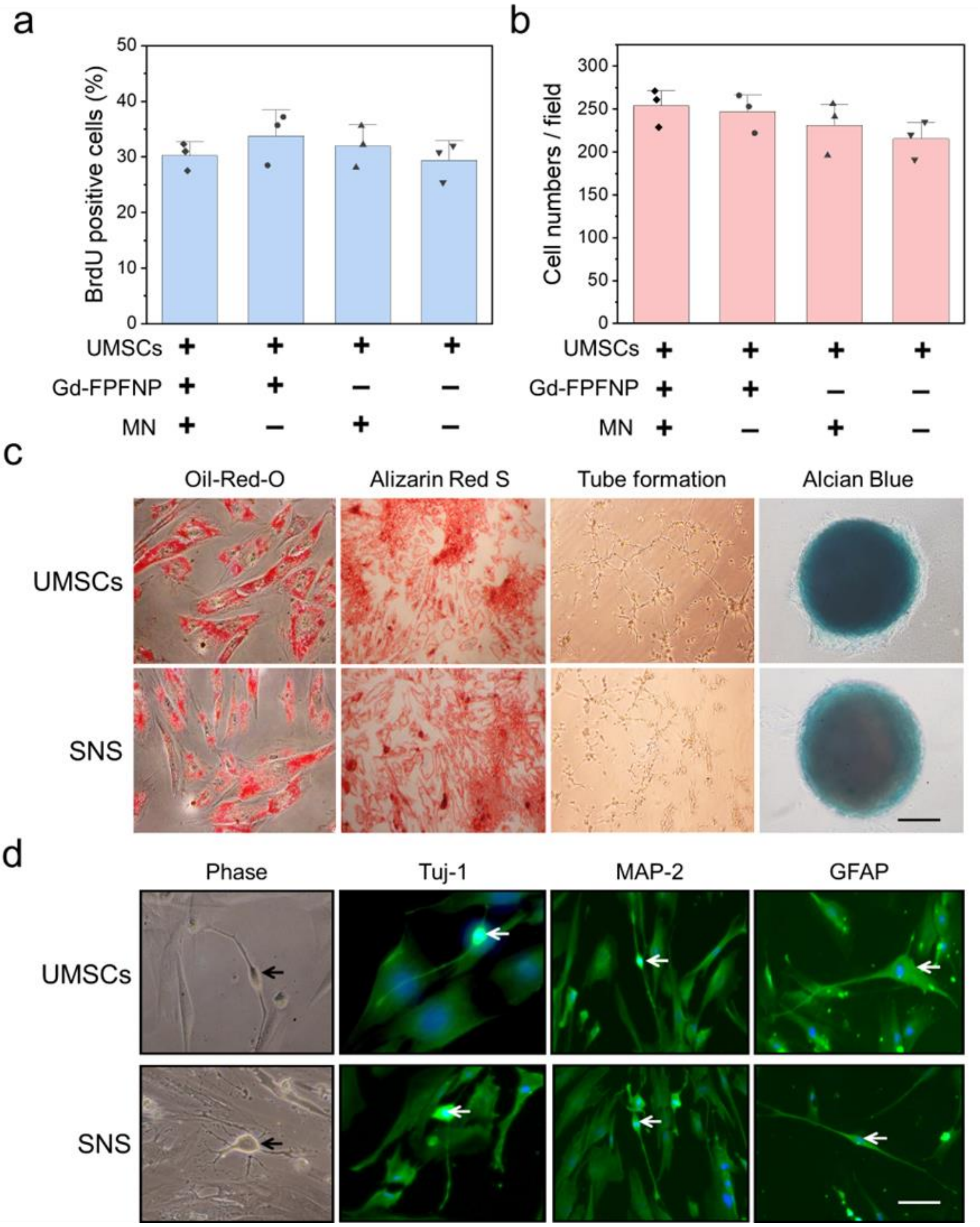

Figure S9. Functional assessment of UMSCs with Gd-FPFNP. Analysis of a) proliferation and b) migration ability of UMSCs under different conditions. c) Adipogenic, chondrogenic, vascular tube formation and osteogenic differentiation ability of UMSCs and SNS. d) Neuroglial cell differentiation of UMSCs and SNS. For c) and d) scale bar = 50 μm. For a) and b), the results were expressed as mean ± SD, n = 3 biologically independent UMSC samples.

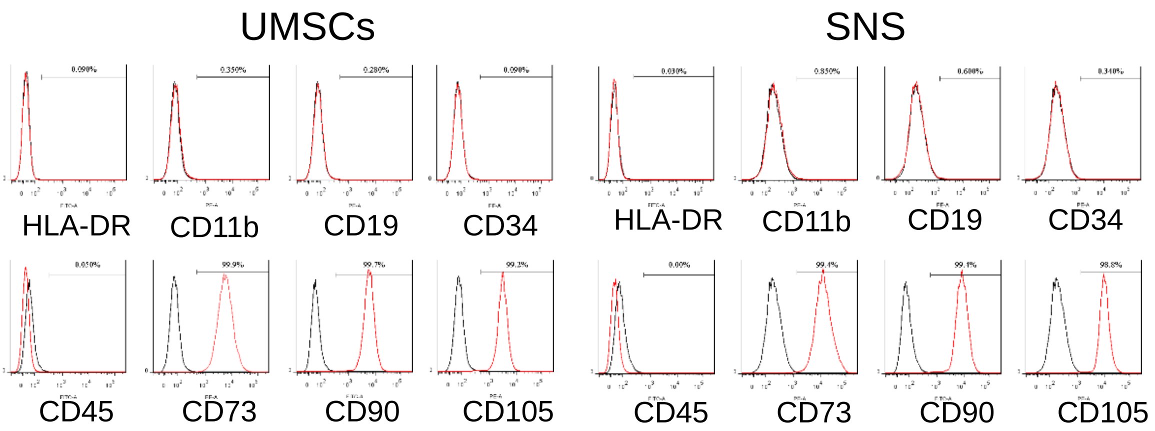

Figure S10. The surface biomarkers of UMSCs and SNS (Gd-FPFNP–treated UMSC).

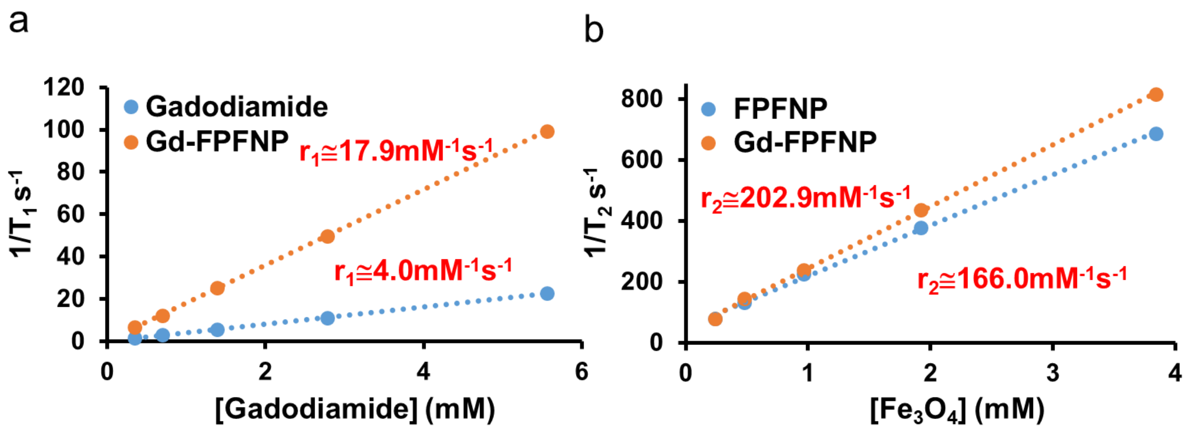

Figure S11. a) MR relaxation rate (r_1) of gadodiamide and Gd-FPFNP at different Gd concentrations. b) MRI relaxation rate (r_2) of FPFNP and Gd-FPFNP at different Fe_3O_4 concentrations.

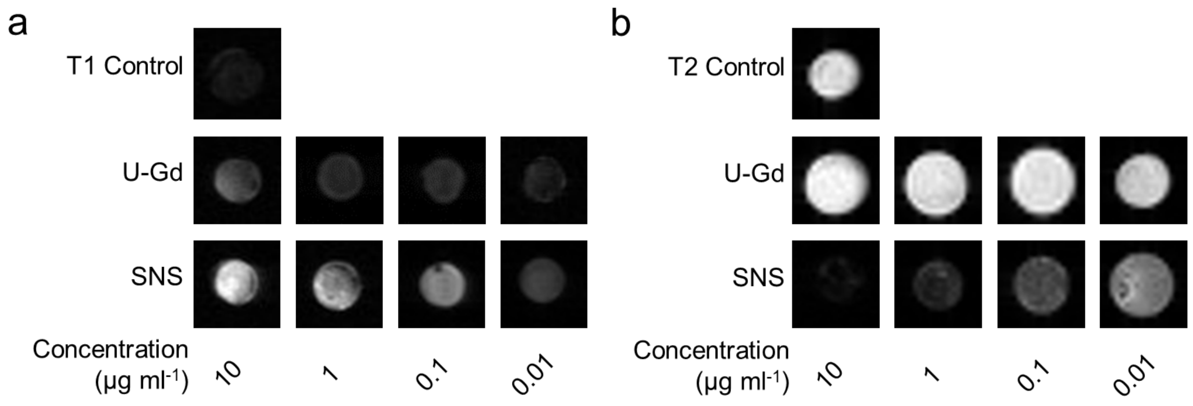

Figure S12. 7-T MRI a) T1 image and b) T2 image of control (agar), gadodiamide plus UMSCs (U-Gd), and SNS.

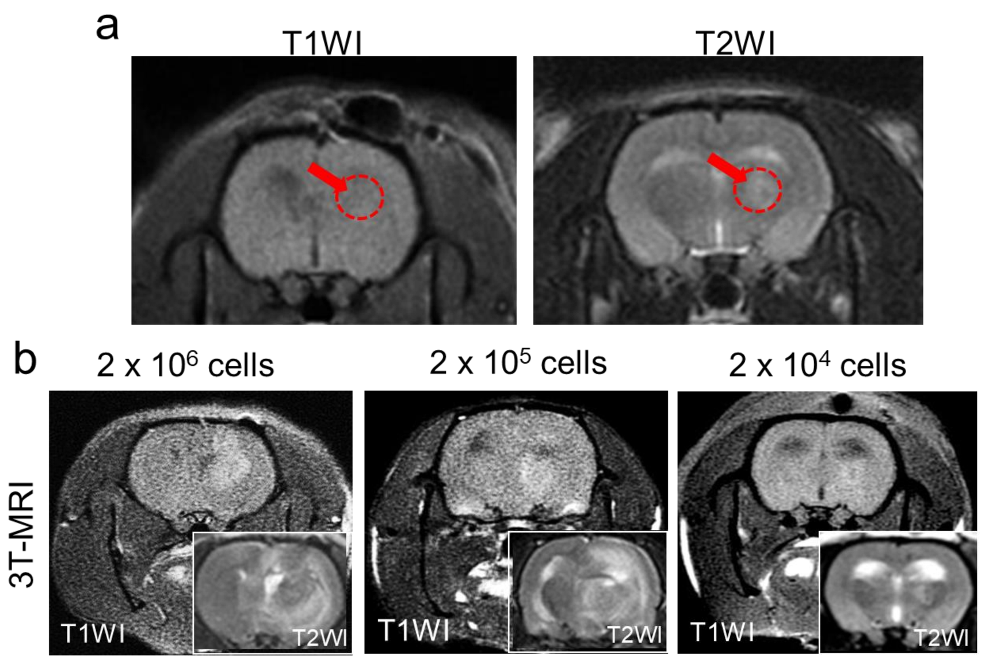

Figure S13. 3T-MRI images of a) pre-contrast rat and b) rats treated with different doses of SNS via intracarotid injection.

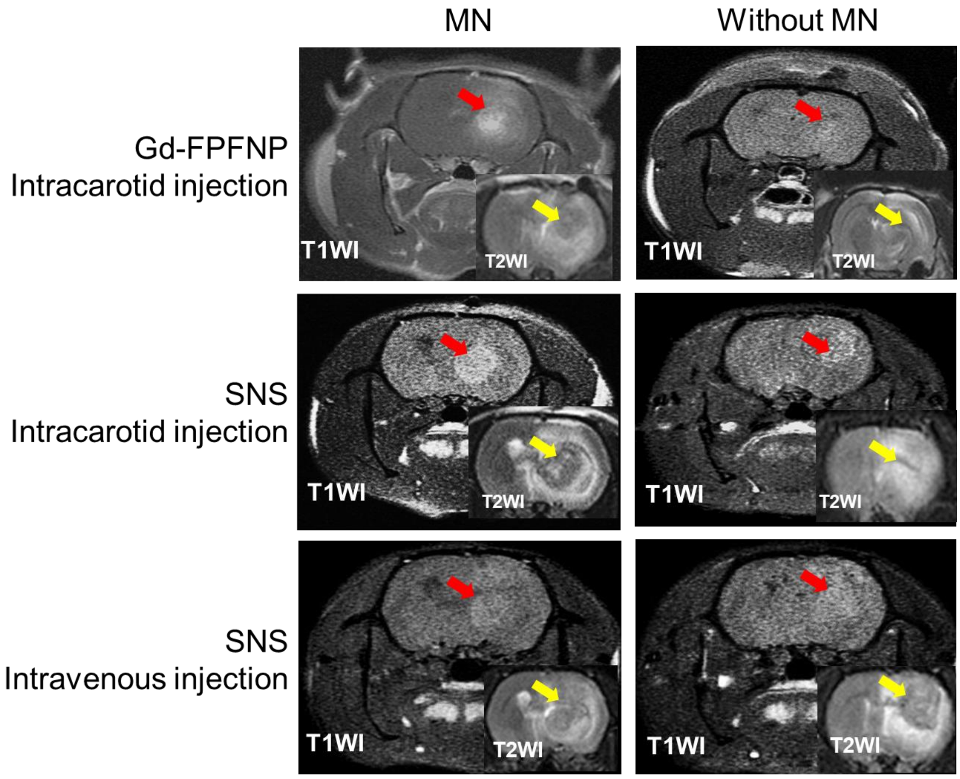

Figure S14. MRI tracking ability. MRI images of F98-Luc rats treated with Gd-FPFNP with/without MN or SNS with/without MN via intracarotid injection, and with SNS with/without MN via intravenous injection.

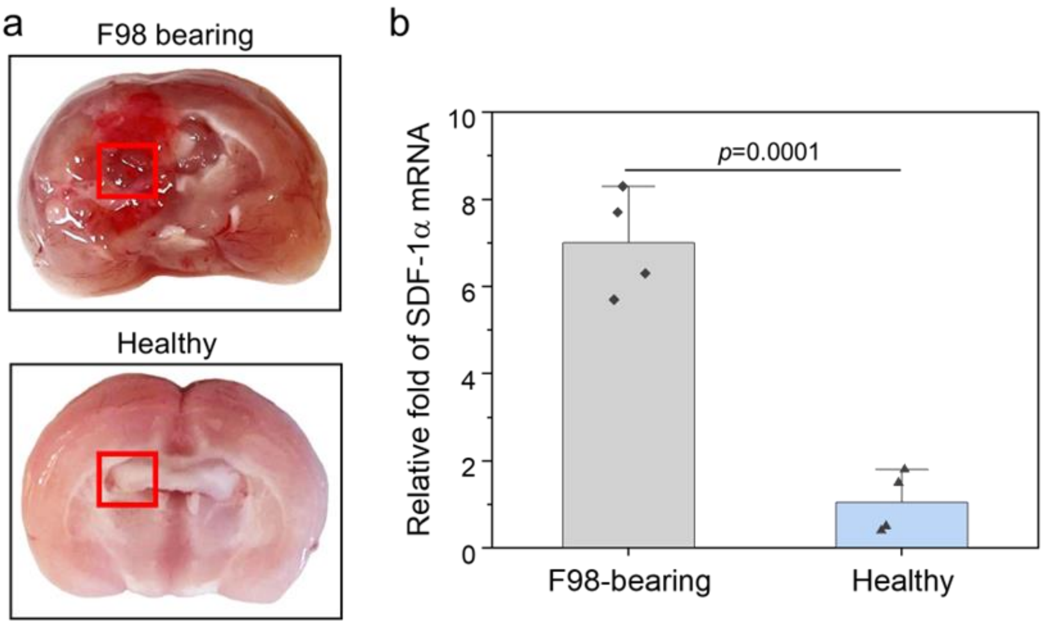

Figure S15. SDF-1 measurement in the brains of tumor-bearing and healthy rats. a) Brain coronal view from rostral to caudal region. The images are representative to 4 images with simialr results. b) The mRNA level of SDF-1α. The results were expressed as mean ± SD, n = 4 rats. Statistical analysis was performed using two-sided t-test. For b) the p value of F98-bearing rats to healthy rats is 0.0001.

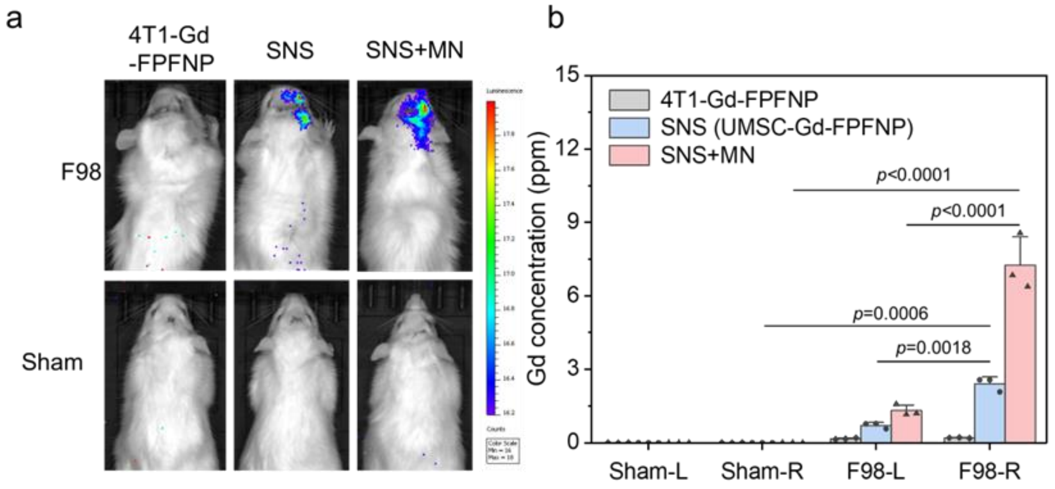

Figure S16. a) The IVIS image of F98-bearing rats and sham rats treated with 4T1-Gd-FPFNP, SNS, or SNS+MN. b) Quantification of the Gd content for the left (L) and right (R) brains of F98-bearing rats and sham rats treated with 4T1-Gd-FPFNP, SNS, or SNS+MN using ICP-MS. Sham-L: left brain of the sham group; Sham-R: right brain of the sham group; F98-L: left brain of the F98-bearing rats; F98-R: right brain of the F98-bearing rats.The results were expressed as mean ± SD, n= 3 rats. Statistical analysis was performed by Graph Pad Prism 9.0 Software: one-way ANOVA with Tukey's multiple comparisons test. For b), the p value of SNS between F98-L and F98-R is 0.0018, SNS plus MN between F98-L and F98-R is < 0.0001, SNS between F98-R and Sham-R is 0.0006, and SNS plus MN between F98-R and Sham-R is < 0.0001.

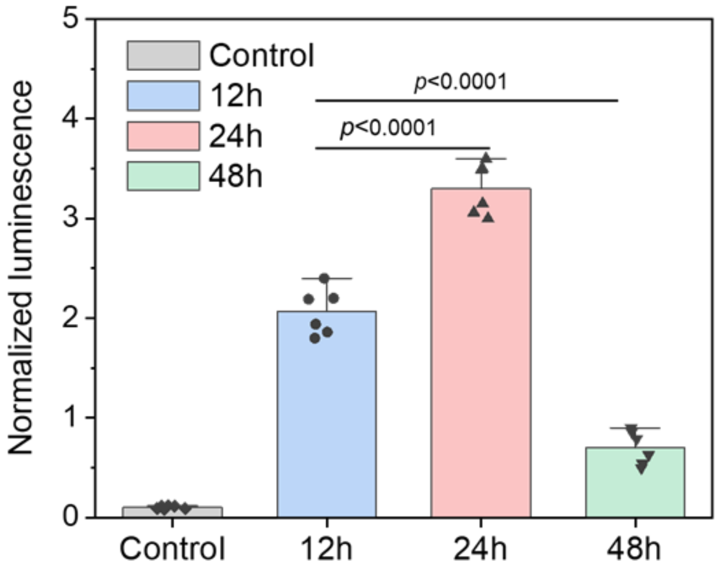

Figure S17. Semi-quantitative data of IVIS in **Figure 3**. The results were expressed as mean ± SD, $n = 6$ rats. Statistical analysis was performed by Graph Pad Prism 9.0 Software: one-way ANOVA with Tukey's multiple comparisons test. The p values of 12h to 24h and 48h are both less than 0.0001.

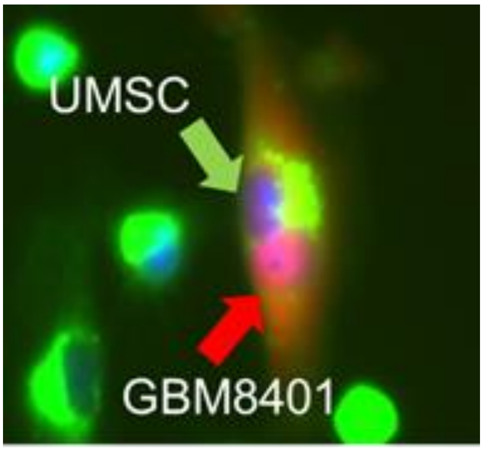

Figure S18. Cell fusion of UMSCs and GBM cells (one cell with two nuclei) after 24 h incubation.

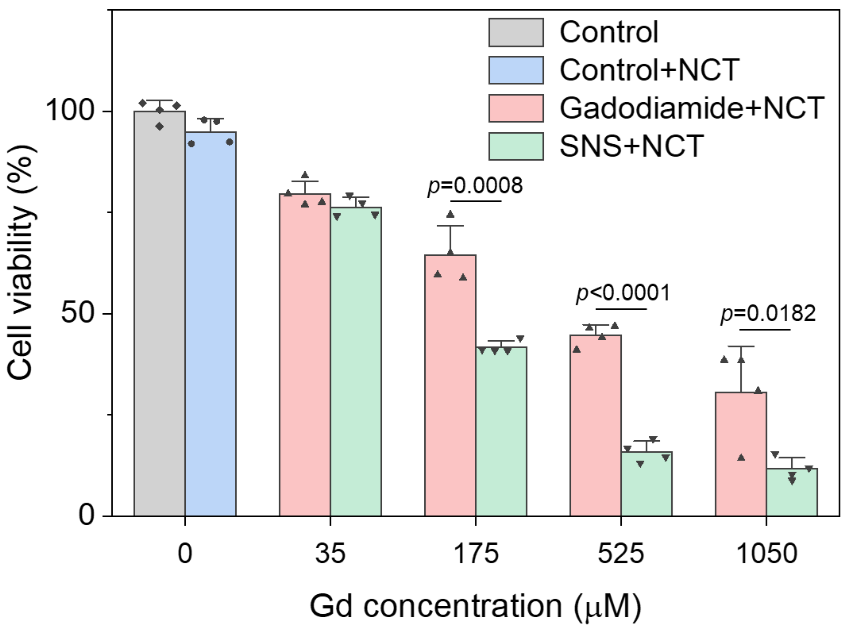

Figure S19. *In vitro* cell viability of GBM8401 (GBM) cells co-cultured with free gadodiamide or SNS under different concentrations of gadodiamide at 24 h post NCT. The results were expressed as mean ± SD, $n = 4$ independent GBM cells. Statistical analysis was performed using two-sided t-test versus free gadodiamide. The p values of SNS plus NCT to gadodiamide plus NCT at Gd concentration of 175, 525, and 1050 μM are 0.0008, < 0.0001, and 0.0182, respectively.

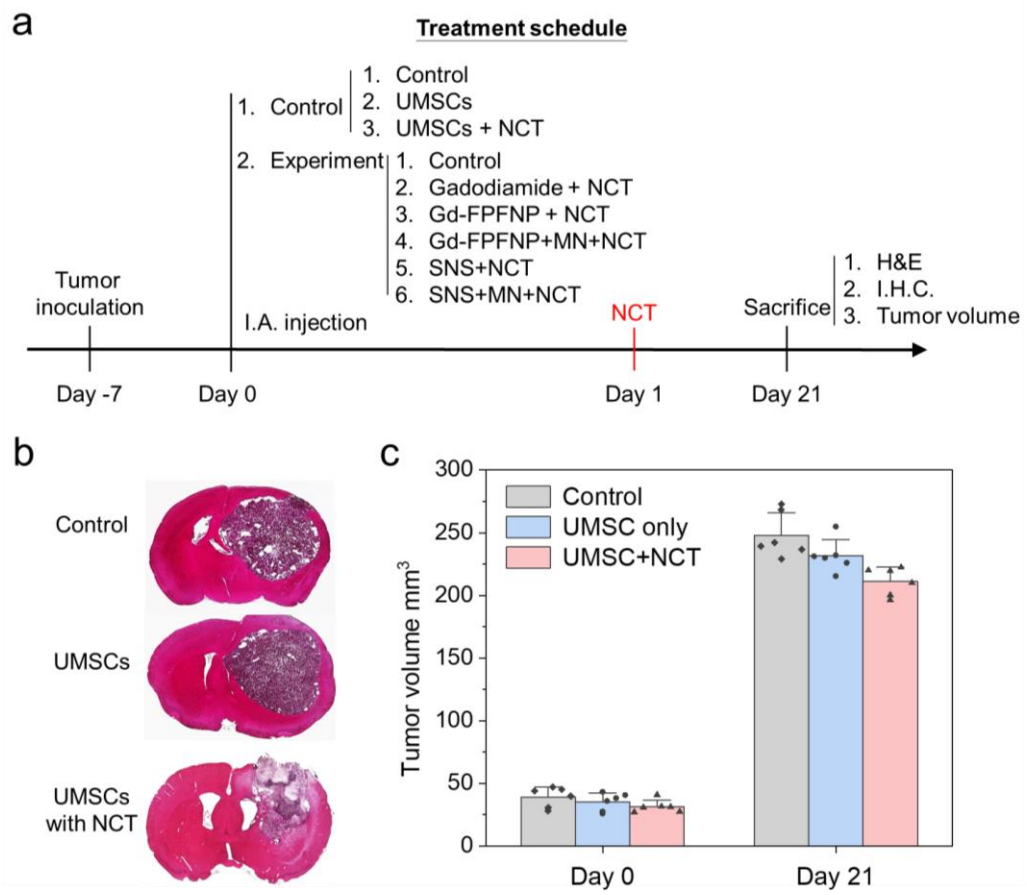

Figure S20. Tumor size after NCT treatment. a) Treatment schedule in our study. b) H&E staining of tumor at 21 days after indicated treatment. c) Tumor volumes at Day 0 (before treatment) and Day 21 (post treatment). The results were expressed as mean ± SD, n = 6 rats.

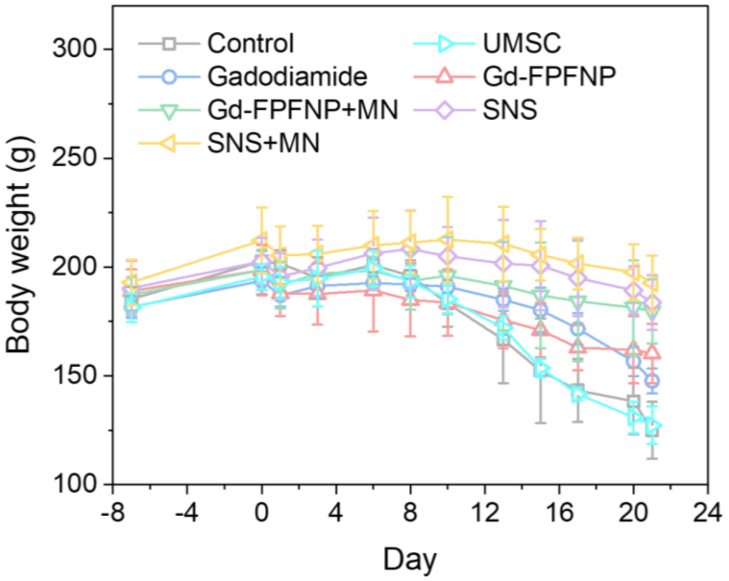

Figure S21. Monitoring of the body weight for the F98-bearing rats treated with saline (control), UMSC, Gadodiamide, Gd-FPFNP, Gd-FPFNP+MN, SNS and SNS+MN plus NCT. The treatment started from Day 0 and the NCT was performed at day 1 (see treatment course plan in Figure S20). The results were expressed as mean ± SD, n = 6 rats.

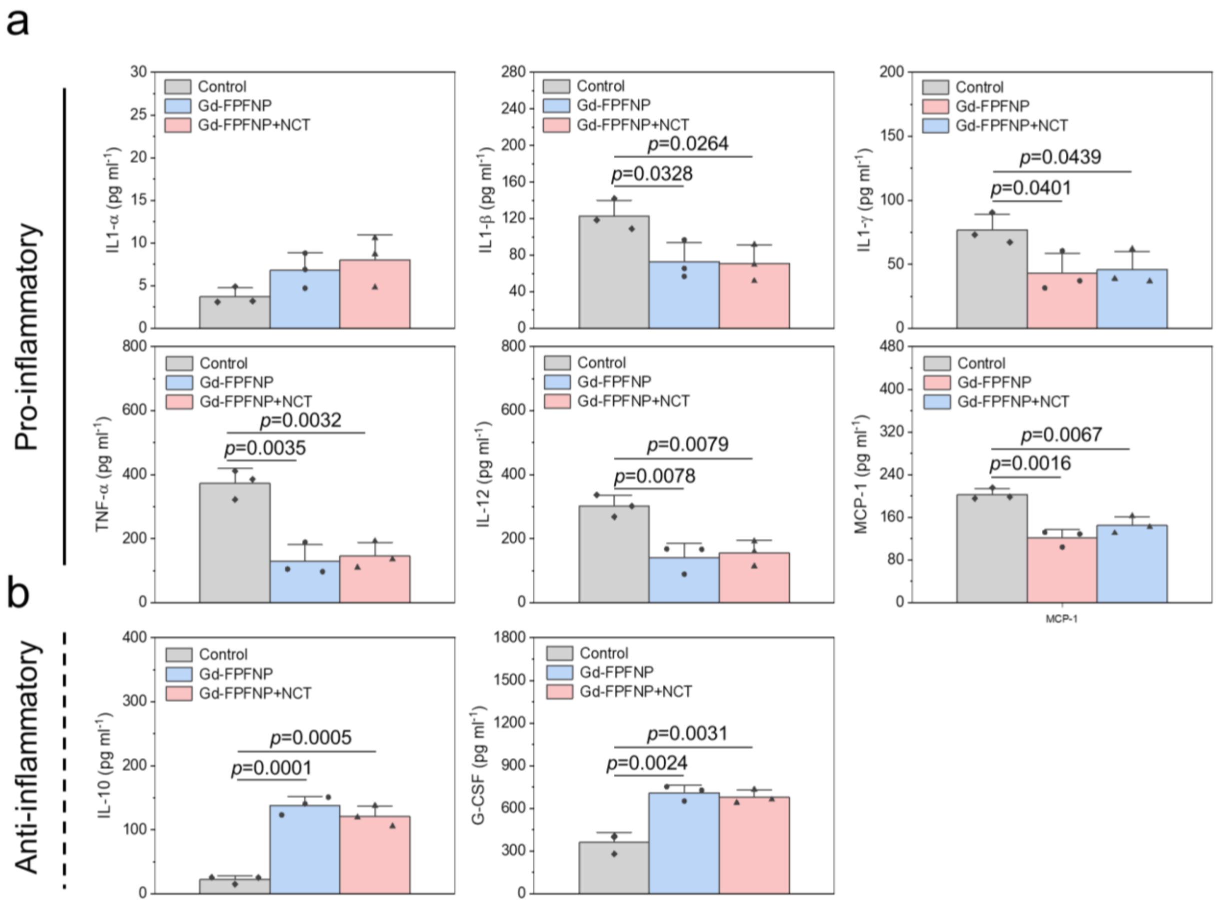

Figure S22. Anti-inflammatory and neuroprotective ability of fucoidan-based nanoparticles. Fucoidan-based nanoparticles mediated immunomodulation-induced neuroprotection in rats bearing GBM. Gd-FPFNP induced a significant reduction in proinflammatory factors in serum: a) IL-1α, IL-1β, IFN-γ, TNF-α, IL-12, MCP-1 and enhancement of anti-inflammatory cytokines: b) IL-10 and G-CSF at 24 h or 48 h after treatment compared to saline control groups. The results are expressed as mean ± SD., n= 3 rats. Statistical analysis was performed by Graph Pad Prism 9.0 Software; two-sided t-test compared with saline-control. The p values between control and Gd-FPFNP and Gd-FPFNP plus NCT are 0.0328 and 0.0264 (IL-1β), 0.0401 and 0.0439 (IFN-γ), 0.0035 and 0.0032 (TNF-α), 0.0078 and 0.0079 (IL-12), 0.0016 and 0.0067 (MCP-1), 0.0001 and 0.0005 (IL-10), 0.0024 and 0.0031 (G-CSF), respectively.

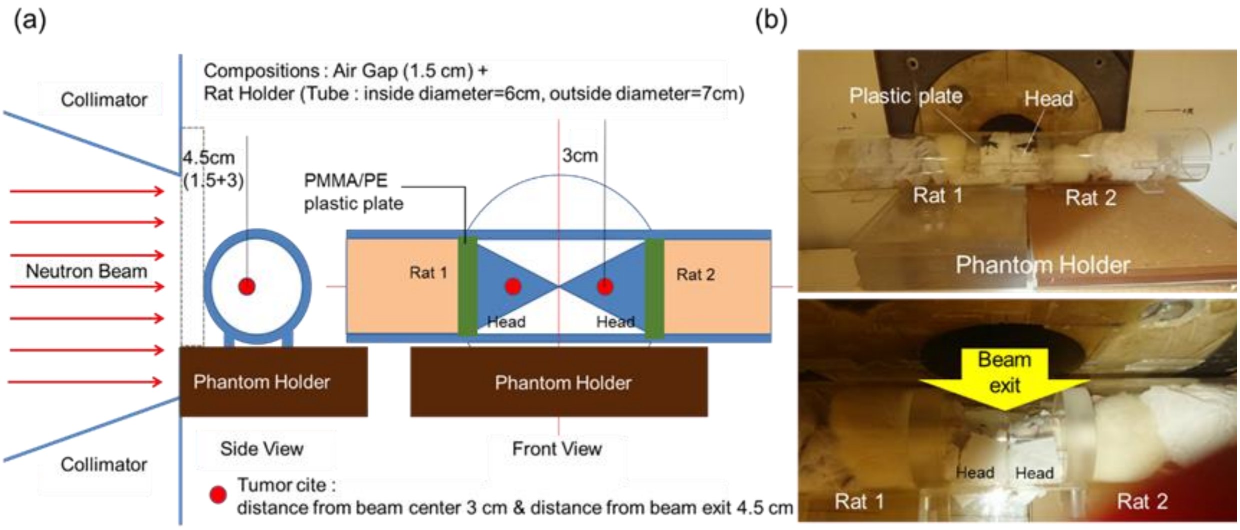

Figure S23. a) Schematic illustration of the relative position of rat holder, rat, tumor site, and the beam exit. b) Representative real image of the relative position of rat holder, rat, tumor site, and the beam exit.

References

1. Nair AB, Jacob S. A simple practice guide for dose conversion between animals and human. *J Basic Clin Pharm* **7**, 27-31 (2016).

2. Hofmann B, Fischer CO, Lawaczeck R, Platzek J, Semmler W. Gadolinium neutron capture therapy (GdNCT) of melanoma cells and solid tumors with the magnetic resonance imaging contrast agent Gadobutrol. *Investigative radiology* **34**, 126-133 (1999).

3. Tokumitsu H, Hiratsuka J, Sakurai Y, Kobayashi T, Ichikawa H, Fukumori Y. Gadolinium neutron-capture therapy using novel gadopentetic acid-chitosan complex nanoparticles: in vivo growth suppression of experimental melanoma solid tumor. *Cancer letters* **150**, 177-182 (2000).

4. Mi P, *et al.* Hybrid Calcium Phosphate-Polymeric Micelles Incorporating Gadolinium Chelates for Imaging-Guided Gadolinium Neutron Capture Tumor Therapy. *ACS Nano* **9**, 5913-5921 (2015).
