# Supplementary material for: Stem cell–nanomedicine system as a theranostic bio-gadolinium agent for targeted neutron capture cancer therapy
Source: Nat Commun. 2023 Jan 18;14:285. doi: 10.1038/s41467-023-35935-0 (PMC9845336; doi:10.1038/s41467-023-35935-0)
Supplement: Supplementary file 2 — Reporting Summary [file 41467_2023_35935_MOESM2_ESM.pdf]

nature portfolio

Corresponding author(s): San-Yuan Chen

Last updated by author(s): Dec 10, 2022

Reporting Summary

Nature Portfolio wishes to improve the reproducibility of the work that we publish. This form provides structure for consistency and transparency in reporting. For further information on Nature Portfolio policies, see our Editorial Policies and the Editorial Policy Checklist.

Statistics

For all statistical analyses, confirm that the following items are present in the figure legend, table legend, main text, or Methods section.

n/a	Confirmed	
☐	☒	The exact sample size (*n*) for each experimental group/condition, given as a discrete number and unit of measurement
☐	☒	A statement on whether measurements were taken from distinct samples or whether the same sample was measured repeatedly
☐	☒	The statistical test(s) used AND whether they are one- or two-sided<br>*Only common tests should be described solely by name; describe more complex techniques in the Methods section.*
☒	☐	A description of all covariates tested
☒	☐	A description of any assumptions or corrections, such as tests of normality and adjustment for multiple comparisons
☐	☒	A full description of the statistical parameters including central tendency (e.g. means) or other basic estimates (e.g. regression coefficient) AND variation (e.g. standard deviation) or associated estimates of uncertainty (e.g. confidence intervals)
☐	☒	For null hypothesis testing, the test statistic (e.g. *F*, *t*, *r*) with confidence intervals, effect sizes, degrees of freedom and *P* value noted<br>*Give P values as exact values whenever suitable.*
☒	☐	For Bayesian analysis, information on the choice of priors and Markov chain Monte Carlo settings
☐	☒	For hierarchical and complex designs, identification of the appropriate level for tests and full reporting of outcomes
☒	☐	Estimates of effect sizes (e.g. Cohen's *d*, Pearson's *r*), indicating how they were calculated

Our web collection on statistics for biologists contains articles on many of the points above.

Software and code

Policy information about availability of computer code

Data collection	No software was used in this study.
Data analysis	Prism 9.0, Origin Pro9.0, Monte Carlo N-particle transport code Version 4C, FlowJo software v.9.3.2

For manuscripts utilizing custom algorithms or software that are central to the research but not yet described in published literature, software must be made available to editors and reviewers. We strongly encourage code deposition in a community repository (e.g. GitHub). See the Nature Portfolio guidelines for submitting code & software for further information.

Data

Policy information about availability of data

All manuscripts must include a data availability statement. This statement should provide the following information, where applicable:
- Accession codes, unique identifiers, or web links for publicly available datasets
- A description of any restrictions on data availability
- For clinical datasets or third party data, please ensure that the statement adheres to our policy

The authors declare that the data supporting the findings of this study are available within the article, source data, and its Supplementary Information. A reporting summary for this article is available as a Supplementary Information file. Source data are provided with this paper.

Field-specific reporting

Please select the one below that is the best fit for your research. If you are not sure, read the appropriate sections before making your selection.

☒ Life sciences ☐ Behavioural & social sciences ☐ Ecological, evolutionary & environmental sciences

For a reference copy of the document with all sections, see nature.com/documents/nr-reporting-summary-flat.pdf

Life sciences study design

All studies must disclose on these points even when the disclosure is negative.

Sample size	Sample sizes were based on previous experience and publications. We preformed sample size calculations on online platform-EDA (Experimental Design Assistant, national centre for the replacement refinement & reduction of animals in research, https://eda.nc3rs.org.uk/about).[1] Following the instructions of EDA, we finished the experiment design diagram and typed the certain parameters which were based on results from published studies for power analysis. This was determined at a minimum of 5 rats for each group.<br>In general, 6-8 rats or 3-4 samples per group are appropriate for in vivo experiments involving therapeutic efficacy, survival, and flow cytometry.<br>1. Towner RA, et al. Regression of glioma tumor growth in F98 and U87 rat glioma models by the Nitrone OKN-007. Neuro-oncology 15, 330-340 (2013). 330-340 (2013).
Data exclusions	No data was excluded.
Replication	As reported in Methods Section, experiments were all replicated at at least three times (n = 3 rats, mice or cell samples cultured in independent space such as plate or disk), and attempts at replication were successful.
Randomization	All animals were randomized between groups.
Blinding	Animals were assigned randomly to control and experimental groups. Although experimenters were not blinded to group allocation for data collection, subsequent offline analysis was performed blinded to experimental conditions. The experimenters were blinded to experimental conditions for analysis of immunohistochemistry and in situ hybridization.

Reporting for specific materials, systems and methods

We require information from authors about some types of materials, experimental systems and methods used in many studies. Here, indicate whether each material, system or method listed is relevant to your study. If you are not sure if a list item applies to your research, read the appropriate section before selecting a response.

Materials & experimental systems

n/a	Involved in the study	
☐	☒	Antibodies
☐	☒	Eukaryotic cell lines
☒	☐	Palaeontology and archaeology
☐	☒	Animals and other organisms
☐	☒	Human research participants
☒	☐	Clinical data
☒	☐	Dual use research of concern

Methods

n/a	Involved in the study	
☒	☐	ChIP-seq
☐	☒	Flow cytometry
☐	☒	MRI-based neuroimaging

Antibodies

Antibodies used	Antibodies: the antibodies used in identifying the stemness of MSC including anti-CD13 (clone WM15, Cat No: 555394), anti-CD29 (clone MAR4, Cat No: 555443), anti-CD44 (clone G44-26, Cat No: 550988), anti-CD73 (clone AD2, Cat No: 550257), anti-CD90 (clone 5E10, Cat No: 559869), anti-CD105 (clone 266, Cat No: 560839, anti-CD166 (clone 3A6, Cat No: 559263), anti-CD49b (clone 12F1, Cat No: 555669), anti-CD1d (clone CD1d42, Cat No:563505), anti-CD3 (clone SK7, 340542), anti-CD10 (clone HI10a, Cat No: 561002), anti-CD14 (clone M5E2, cat No: 561712), anti-CD31 (clone MEC13.3, cat No: 550274), anti-CD34 (clone 581, cat No: 555824), anti-CD45 (clone HI30, 560975), anti-CD49d (clone 9F10, cat No: 560972), anti-CD56 (clone B159, cat No: 555518), anti-CD117 (clone 104D2, cat No: 340529), anti-HLA-ABC (clone G46-2.6, cat No: 555555), and anti-HLA-DR (clone G46-6, cat No: 560944) were purchased from BD Biosciences and were all diluted at 1:100. For the antibodies used in immunocytochemical assessment including the ones against luciferase (clone21, diluted at 1:200), GFAP (clone GA5, diluted at 1:1000), Tuj-1 (clone TU-20, diluted at 1:1000), or MAP-2 (clone AP20, diluted at 1:300) were purchased from Millipore. Anti-β-Actin (clone mAbcam 8226, diluted at 1:1000) was purchased from Abcam.
Validation	Antibody validation is based on previously published data.<br>anti-CD13 (clone WM15, Cat No: 555394), https://www.bdbiosciences.com/en-us/products/reagents/flow-cytometry-reagents/research-reagents/single-color-antibodies-ruo/pe-mouse-anti-human-cd13.555394

anti-CD29 (clone MAR4, Cat No: 555443), https://www.bdbiosciences.com/en-sg/products/reagents/flow-cytometry-reagents/research-reagents/single-color-antibodies-ruo/pe-mouse-anti-human-cd29.555443
anti-CD44 (clone G44-26, Cat No: 550988), https://www.bdbiosciences.com/en-us/products/reagents/flow-cytometry-reagents/research-reagents/single-color-antibodies-ruo/purified-mouse-anti-human-cd44.550988
anti-CD73 (1:100, clone AD2, Cat No: 550257), https://www.bdbiosciences.com/en-us/products/reagents/flow-cytometry-reagents/research-reagents/single-color-antibodies-ruo/pe-mouse-anti-human-cd73.550257
anti-CD90 (1:100, clone 5E10, Cat No: 559869), https://www.bdbiosciences.com/en-us/products/reagents/flow-cytometry-reagents/research-reagents/single-color-antibodies-ruo/apc-mouse-anti-human-cd90.559869
anti-CD105 (1:100, clone 266, Cat No: 560839), https://www.bdbiosciences.com/en-us/products/reagents/flow-cytometry-reagents/research-reagents/single-color-antibodies-ruo/pe-mouse-anti-human-cd105.560839
anti-CD166 (1:100, clone 3A6, Cat No: 559263), https://www.bdbiosciences.com/en-us/products/reagents/flow-cytometry-reagents/research-reagents/single-color-antibodies-ruo/pe-mouse-anti-human-cd166.559263
anti-CD49b (1:100, clone 12F1, Cat No: 555669), https://www.bdbiosciences.com/en-au/products/reagents/flow-cytometry-reagents/research-reagents/single-color-antibodies-ruo/pe-mouse-anti-human-cd49b.555669
anti-CD1d(1:100, clone CD1d42, Cat No: 563505), https://www.bdbiosciences.com/en-us/products/reagents/flow-cytometry-reagents/research-reagents/single-color-antibodies-ruo/apc-mouse-anti-human-cd1d.563505
anti-CD3 (1:100, clone SK7, Cat No: 340542), https://www.bdbiosciences.com/en-nz/products/reagents/flow-cytometry-reagents/clinical-diagnostics/single-color-antibodies-asr-ivd-ce-ivd/cd3-fitc.340542
anti-CD10 (clone HI10a, Cat No: 561002), https://www.bdbiosciences.com/en-us/products/reagents/flow-cytometry-reagents/research-reagents/single-color-antibodies-ruo/pe-mouse-anti-human-cd10.561002
anti-CD14 (clone M5E2, cat No: 561712), https://www.bdbiosciences.com/en-nz/products/reagents/flow-cytometry-reagents/research-reagents/single-color-antibodies-ruo/fitc-mouse-anti-human-cd14.561712
anti-CD31 (clone MEC13.3, cat No: 557508), https://www.bdbiosciences.com/en-au/products/reagents/flow-cytometry-reagents/research-reagents/single-color-antibodies-ruo/fitc-mouse-anti-human-cd31.557508
anti-CD34 (clone 581, cat No: 555824), https://www.bdbiosciences.com/en-us/products/reagents/flow-cytometry-reagents/research-reagents/single-color-antibodies-ruo/apc-mouse-anti-human-cd34.555824
anti-CD45 (clone HI30, 560975), https://www.bdbiosciences.com/en-us/products/reagents/flow-cytometry-reagents/research-reagents/single-color-antibodies-ruo/pe-mouse-anti-human-cd45.560975
anti-CD49d (clone 9F10, cat No: 560972), https://www.bdbiosciences.com/en-ca/products/reagents/flow-cytometry-reagents/research-reagents/single-color-antibodies-ruo/pe-mouse-anti-human-cd49d.560972
anti-CD56 (clone B159, cat No: 555518), https://www.bdbiosciences.com/en-us/products/reagents/flow-cytometry-reagents/research-reagents/single-color-antibodies-ruo/apc-mouse-anti-human-cd56-ncam-1.555518
anti-CD117 (clone 104D2, cat No: 340529), https://www.bdbiosciences.com/en-au/products/reagents/flow-cytometry-reagents/clinical-discovery-research/single-color-antibodies-ruo-gmp/pe-mouse-anti-human-cd117.340529
anti-HLA-ABC (clone G46-2.6, cat No: 555555), https://www.bdbiosciences.com/en-us/products/reagents/flow-cytometry-reagents/research-reagents/single-color-antibodies-ruo/apc-mouse-anti-human-hla-abc.555555
anti-HLA-DR (clone G46-6, cat No: 560944), https://www.bdbiosciences.com/en-us/products/reagents/flow-cytometry-reagents/research-reagents/single-color-antibodies-ruo/fitc-mouse-anti-human-hla-dr.560944
anti-luciferase (clone21, Millipore), https://www.merckmillipore.com/TW/zh/product/Anti-Luciferase-Antibody-clone21,MM_NF-05-603
anti-GFAP (clone GA5, Millipore), https://www.merckmillipore.com/TW/zh/product/Anti-Glial-Fibrillary-Acidic-Protein-Antibody-clone-GA5,MM_NF-MAB3402
anti-Tuj-1 (clone TU-20, Millipore), https://www.merckmillipore.com/TW/zh/product/Anti-Tubulin-Antibody-beta-III-isoform-CT-clone-TU-20-Similar-to-TUJ1,MM_NF-MAB1637
anti-MAP-2 (clone AP20, Millipore), https://www.merckmillipore.com/TW/zh/product/Anti-MAP2-Antibody-clone-AP20,MM_NF-MAB3418
anti-β-Actin (clone mAbcam 8226, Abcam), https://www.abcam.com/beta-actin-antibody-mabcam-8226-loading-control-ab8226.html
Validation of the antibodies was according to the website of manufacturing company or was achieved using flow cytometry and IHC staining.

Eukaryotic cell lines

Policy information about cell lines

Cell line source(s)	F98 ((ATCC® CRL-2397™), GBM 8401(BCRC Number: 60163), 4T1(ATCC® CRL-2539™), UMSCs (The collected human umbilical cord tissues approved by the Institutional Review Board (IRB) of the China Medical University Hospital in Taichung (ROC)).
Authentication	The cell lines were authenticated by the supplier using morphology, karyotyping, and PCR-based approaches.
Mycoplasma contamination	We did not detect contamination of the cells using Hoechst DNA stain method and agar culture method.
Commonly misidentified lines (See ICLAC register)	Not any misidentified line is used in this study.

Animals and other organisms

Policy information about studies involving animals; ARRIVE guidelines recommended for reporting animal research

Laboratory animals	The female rats strain F344/NNarl (RMRC21002) aged 8 weeks and the male mice strain C57BL/6JNarl (RMRC11005) aged 8 weeks both were purchased from National Laboratory Animal Center, Taipei, Taiwan.

	Housing condition: temperature:23±2°C; humidity: 50±10%; dark/light cycle: 12hour/12hour.
Wild animals	Not any wild animal was used in this study.
Field-collected samples	The study did not involve samples collected from the field.
Ethics oversight	National Animal Center of Taiwan, Ministry of Science and Technology (ROC), and Instituitional Committee of Animal Research of China Medical University approved the animal experiments in this study under IACUC number CMU 2020-014.

Note that full information on the approval of the study protocol must also be provided in the manuscript.

Human research participants

Policy information about studies involving human research participants

Population characteristics	To collect umbilical cord mesenchymal stem cells, 30 pregnant women were designed to be included in the studies for the placenta donation. The inclusion criteria were (1) 34 weeks or longer pregnant with intact placenta, (2) free of complications of pregnancy, and (3) healthy pregnant women and fetus. The exclusion criteria were (1) with critical illness such as diabetes, immune-related diseases, or cancers, (2) asymptomatic hepatitis A,B or C carriers and syphilis patients, (3) being evaluated as an improper case by the doctor, (4) having or have even been diagnosed with pulmonary tuberculosis, and (5) tested positive with treponema pallidum, Chlamydia trachomatis, human T cell leukemia virus, West Nile Virus or Neisseria gonorrhea within 8 weeks prior to giving birth, and (6) ever being diagnosed withs Zika or HIV.
Recruitment	Written informed consent was acquired from the pregnant women. The pregnant women who agree to donate the placenta and match the above characteristic were approached and recruited into the studies without any additional bias.
Ethics oversight	IRB: #CMUH-110-REC-1-068

Note that full information on the approval of the study protocol must also be provided in the manuscript.

Flow Cytometry

Plots

Confirm that:

- [x] The axis labels state the marker and fluorochrome used (e.g. CD4-FITC).
- [x] The axis scales are clearly visible. Include numbers along axes only for bottom left plot of group (a 'group' is an analysis of identical markers).
- [x] All plots are contour plots with outliers or pseudocolor plots.
- [x] A numerical value for number of cells or percentage (with statistics) is provided.

Methodology

Sample preparation	Sample preparation was described at the "Method" part in manuscript.
Instrument	BD FACSCanto II (#338962 FACSCanto Flow Cytometer)
Software	FACSDiva software v9.3.2
Cell population abundance	The umbilical cord explants were cultured in DMEM containing 10% fetal calf serum (FCS, Gibco) and antibiotics at 37 °C in a 95% air/5% CO2 humidified atmosphere. They were left undisturbed for 5–7 days to allow for migration of the cells from the explants. The cellular morphology of umbilical cord-derived mesenchymal stem cells (UMSCs) became homogenously spindle shaped in cultures after 4–8 passages as shown in Figure S4, and the specific surface molecules of cells from the WJ were characterized by flow cytometric analysis. The cells were detached with 2 mM EDTA in PBS, washed with PBS containing 2% BSA and 0.1% sodium azide (Sigma, USA) and incubated with the respective antibody conjugated with fluorescein isothiocyanate (FITC) or phycoerythrin (PE) including CD13, CD29,CD44,CD73,CD90, CD105,CD166, CD49b, CD1d, CD3, CD10, CD14, CD31, CD34, CD45, CD49d, CD56, CD117, HLA-ABC, and HLA-DR (BD, PharMingen).
Gating strategy	The gating strategies was performed based on the justification of first gate, exclusion of doublets by FSC-A and FSC-H, exclusion of dead cells by selection of 7-AAD-/CD90+

- [x] Tick this box to confirm that a figure exemplifying the gating strategy is provided in the Supplementary Information.

Magnetic resonance imaging

Experimental design

Design type	To evaluate the targeting ability and accumulation of SNS in GBM via the signal from gadodiamide and nano iron in Gd-FPFNP (In vivo). To evaluate the imaging ability of SNS (In vitro).
Design specifications	After injection of different groups as figure 4 listed, MRI was performed on F344 rats under anesthesia in a 3-Tesla (3-T)

MRI scanner (GE) at various time points (12 h, 24 h, and 48 h) after cells transplantation on animals under anesthesia in 3-Tesla (GE) MRI scanner. For each rat, we acquired 22 coronal and axial images with 0.7 mm thick slices.
MSC cells were seeded in 6-well culture plates. The cells were added with different Gd concentrations (10, 1, 0.1, 0.01ug/ml) with or without nanoparticles and were then dispersed in 2% agarose. The images were performed using a 7-Tesla (7-T) PharmaScan (Bruker).

Behavioral performance measures

No behavioural performance was measured.

Acquisition

Imaging type(s)

T1-weighted and T2-weighted imaging for the cross sections of the rat brain.

Field strength

3-Tesla (In vivo). 7-Tesla (In vitro).

Sequence & imaging parameters

In vivo:
For spin-echo (SE) T1-weighted imaging (T1WI), the images were acquired with repetition time/echo time (TR/TE) of 500/15 ms, field of view (FOV) of 28×44 mm. Rapid Acquisition with Relaxation Enhancement (RARE) spin echo sequence was used for fast T2-weighted imaging (T2WI, TE 50ms, TR 3000ms) with a 256 ×256 in-plane matrix and a 2.56cm field of view (FOV).

In vitro:
T1-weighted images were obtained by T1-FLASH sequences as follows: TE=9 ms, TR= 500 ms, matrix size=256*256 and NEX=16. T2-weighted images were obtained by T2 RARE sequences as follows: TE=19 ms, TR=3000 ms, matrix size=256*256 and NEX=1.

Area of acquisition

Whole brain.

Diffusion MRI ☐ Used ☒ Not used

Preprocessing

Preprocessing software

No preprocessing was used.

Normalization

No normalization was used.

Normalization template

No template was used.

Noise and artifact removal

No noise removal or artifact removal were used

Volume censoring

No volume censoring was used.

Statistical modeling & inference

Model type and settings

No statistical modeling was used.

Effect(s) tested

No statistical effects were tested.

Specify type of analysis: ☒ Whole brain ☐ ROI-based ☐ Both

Statistic type for inference
(See Eklund et al. 2016)

None used.

Correction

None needed.

Models & analysis

n/a	Involved in the study
☒	☐ Functional and/or effective connectivity
☒	☐ Graph analysis
☒	☐ Multivariate modeling or predictive analysis
